# Olfaction with legs—Spiders use wall-pore sensilla for pheromone detection

**DOI:** 10.1073/pnas.2415468121

**Published:** 2025-01-06

**Authors:** Mohammad Belal Talukder, Carsten H. G. Müller, Dan-Dan Zhang, Stefan Schulz, Christer Löfstedt, Hong-Lei Wang, Gabriele B. Uhl

**Affiliations:** ^a^General and Systematic Zoology, Zoological Institute and Museum, University of Greifswald, Greifswald 17489, Germany; ^b^Pheromone Group, Department of Biology, Lund University, Lund 22362, Sweden; ^c^Chemical Ecology, Institute of Organic Chemistry, Technische Universität Braunschweig, Braunschweig 38106, Germany

**Keywords:** chemosensing, ultrastructure, electrophysiology, mate attraction, Araneae

## Abstract

The sense of smell is a central sensory modality of most terrestrial species. However, our knowledge of olfaction is based on vertebrates and insects. In contrast, little is known about the chemosensory world of spiders and nothing about how they perform olfaction despite their important ecological role. The orb-weaving spider *Argiope bruennichi* lends itself to an in-depth study on olfaction as it is one of the few spider species whose volatile sex pheromone, emitted by females to attract males, is known. We combined ultrastructural and electrophysiological analyses and found that previously overlooked sensilla with wall pores are abundant on all walking legs of *A. bruennichi* males. We compared the ultrastructure of these wall-pore sensilla with those known to perform olfaction in insects, exploring similarities and differences. Electrophysiological single sensillum recordings demonstrated that the wall-pore sensilla in *A. bruennichi* respond highly sensitive and in a concentration-dependent manner to the sex pheromone. Our study demonstrates male-specific sensilla for detecting signaling females, whereas females and subadult males are devoid of wall pore sensilla. In a preliminary comparative morphological analysis using 19 species from 16 spider families, we found that wall-pore sensilla occur in male spiders from most, but not in basally branching clades or in Salticids, suggesting that wall-pore sensilla evolved at least once within spiders and were lost at least once. This research significantly expands our knowledge of the sensory ecology of spiders, will stimulate studies on the diversity and function of sensilla, as well as studies on the evolution of olfaction in arthropods.

## Significance

Communication via sex pheromones is well studied in insects. Spiders are the most important group of natural enemies of insects, but we do not know much about their chemosensory world. Here, we provide evidence that males of the orb-weaver spider *Argiope bruennichi* perceive the sex pheromone produced by female conspecifics using sensilla that possess wall pores, similar to those known from insects. The sensilla are found in male *A. bruennichi* but not in females or subadult males, which strongly suggests that their main function is related to species discrimination, mate search, and mate assessment. We did not find wall-pore sensilla in the basally branching spider lineages, suggesting that olfactory sensilla evolved several times independently within arachnids.

Chemical sensing is fundamental in all living organisms. Arthropods convert chemical stimuli into bioelectric signals through specialized receptors in sensory hairs called sensilla. Studies on chemosensing in insects have shown that contact chemoreception, i.e., taste, is primarily performed by sensilla that have a single pore at the tip (tip-pore sensilla, tp-sensilla), while airborne volatile chemicals are detected by sensilla that have multiple pores in the wall of their shaft (wall-pore sensilla, wp-sensilla). In both types of sensilla, chemicals pass through the pore(s) and reach the dendrites of receptor cells that run within the sensillum shaft (1, 2). While tp-sensilla have been reported for many spider species (3–5), there is only one report of the ultrastructure of a wp-sensillum in a single specimen of a New Zealand *Gradungula* spider (3). Consequently, the structural and functional basis of how spiders perform olfaction has remained unsolved despite a wealth of behavioral data demonstrating that spiders emit and respond to chemical signals, allowing them to detect the presence of prey and predators, and locate mates from considerable distances (6–10).

To test for the presence of olfactory sensilla, we chose the wasp spider *Argiope bruennichi* as our focal species. The mating behavior of *A. bruennichi* has been extensively studied, and males are shown to be attracted to webs of virgin females from a distance (11, 12). The pheromone responsible for mate attraction was identified as a methyl citrate ester, namely a mixture of the 2*R*,3*S*- and 2*S*,3*S*-isomers of trimethyl methylcitrate (8). Using synthetic compounds in a bioassay corroborated the biological activity of the methylcitrate—males were attracted to pheromone traps in the field in a concentration-dependent manner (8). As to the morphological equipment for chemosensing, tp-sensilla were found in high numbers in *A. bruennichi* and occur mainly on the distal segments of the walking legs and pedipalps of both sexes, where contact with the substrate is most likely (5, 13). That tp-sensilla respond to the species-specific contact pheromone was demonstrated in another spider, *Cupiennius salei,* using electrophysiological recordings (4). In the search of olfactory sensilla, we reexamined all sensilla on the walking legs and pedipalps of *A. bruennichi* and defined areas that do not come into contact with the substrate as candidate areas for olfactory sensilla (SI Appendix, *Supporting Text*, Fig. S1, and Table S1 and Movie S1). We found wp-sensilla and that they are abundant in *A. bruennichi* males in noncontact areas of all walking legs and demonstrated their responsiveness to the female sex pheromone. These sensilla do not occur in females and subadult males. The morphological inspection of sensilla in 19 other spider species from different clades suggests that wp-sensilla evolved within spiders, and likely several times convergently in arachnids.

## External Anatomy of Candidate Olfactory Sensilla in Noncontact Areas

**Fig. 1.**
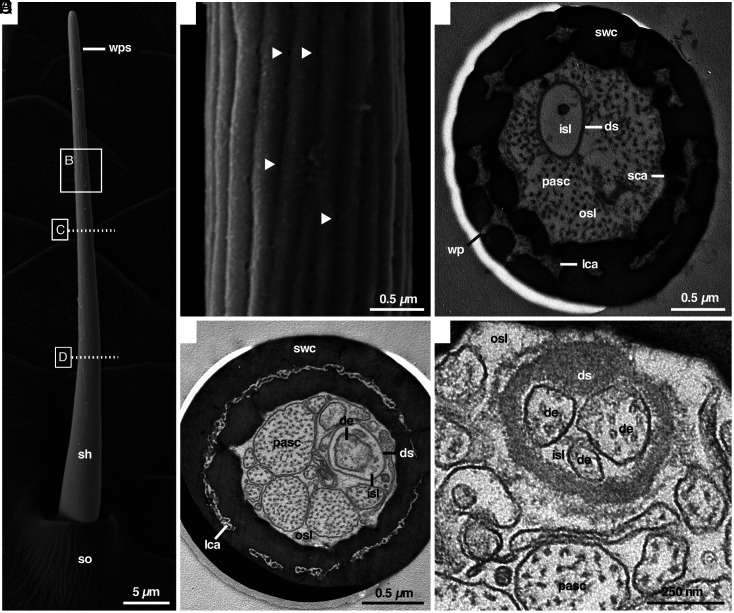
External appearance and cross-sections of wall-pore sensilla in *A. bruennichi* males. (*A*) SEM-overview of a wall-pore sensillum (*wps*) with socket (*so*) and a blunt tip. Note the smooth nonporous base of the sensillum shaft (*sh*). (*B*) Sensillum shaft showing wall pores (some with arrowheads) in the grooved region. (*C*) TEM cross-section of the median region of the sensillum shaft shows that wall pores (*wp*) are connected to the longitudinal shaft canals *(lca),* as well as to the outer sensillum lymph space (*osl)* inside the sensillum. (*D*) Wall pores are absent at the basal region of the sensillum, but the longitudinal shaft canals (*lca*) are present throughout the shaft. Dendrites (*de*) are confined to the inner sensillum lymph space (*isl*) surrounded by the dendritic sheath (*ds*). (*E*) Close-up showing three dendrites in the inner sensillum lymph space, surrounded by the continuous dendritic sheath. Further labels; *pasc* processes of accessory sheath cells, *sca* spoke canal, *swc* shaft-wall cuticle.

Using high-resolution scanning electron microscopy (field emission, FE-SEM), we examined the outer structure of all sensilla on the walking legs and pedipalps of male and female *A. bruennichi*. Spiders possess a wealth of hair-like sensilla, including various types of mechanosensilla (no pores and flexible sensillum shaft arising from a socket) and tp-sensilla (terminal pore, steep angle, s-shaped shaft arising from a socket) (5). We found small sensilla that possess a socket and a blunt tip without a terminal pore, appearing to be mechanosensilla (Fig. 1*A*). These sensilla possess longitudinal grooves on the shaft that start at about one-fourth of their length. Using FE-SEM, roundish invaginations became visible in these grooves, which we considered as candidates for pore openings [diameter 40.48 ± 3.03 nm, (arithmetic mean ± SD); N = 93] (Fig. 1*B*). The density of the suspected pores on the sensillum shaft is high at 8.96 ± 2.22 pores per µm^2^ (N = 31). We found these candidate wp-sensilla on all walking legs (SI Appendix, Fig. S2), but not on the pedipalps of *A. bruennichi* males. They are slightly curved and relatively short (length 41.62 ± 4.99 µm; N = 31), slender (measured 25 µm above the hair base; diameter 1.44 ± 0.07 µm; N = 31), and oriented at a flat angle almost parallel to the leg axis pointing toward the tip of the leg. These candidate wp-sensilla did not differ noticeably in size depending on their location on the male’s walking legs. Interestingly, we did not find these sensilla in subadult males, nor did we detect similar sensilla or other types of sensilla with possible wall pores in females (SI Appendix, Figs. S3 *A*, C, and *E*, S4 A, *C*, and E, and S5 *A* and B).

## Internal Anatomy of Candidate Wall-Pore Sensilla in Males

**Fig. 2.**
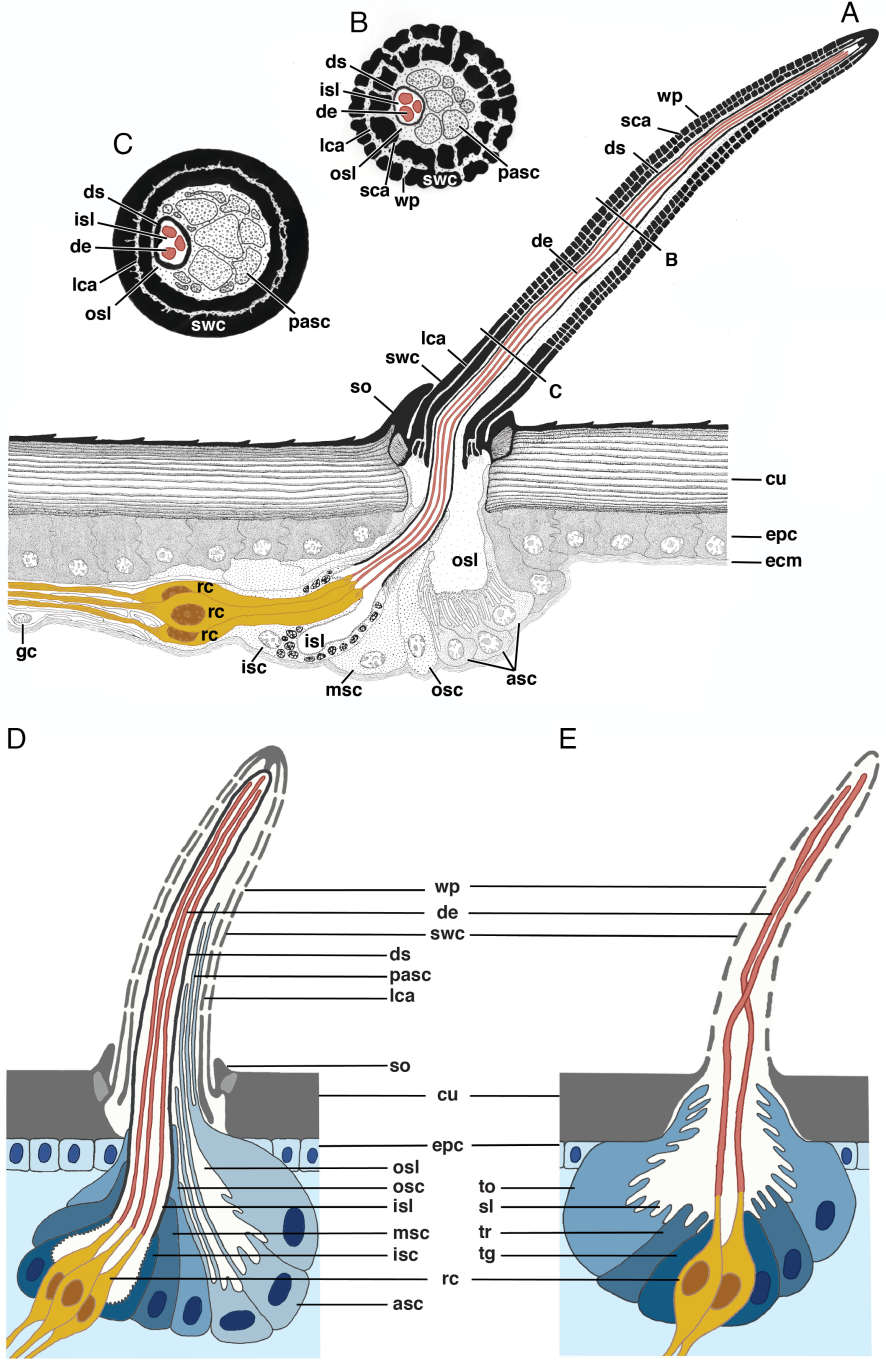
Semischematic (*A*–*C*) and schematic (*D*) reconstruction of a wall-pore sensillum on a walking leg of an *A. bruennichi* male and of a wp-sensillum of an insect (*E*). (*A*) Overview of cellular anatomy in longitudinal view. Sensilla are innervated by 1 to 4 (here 3) receptor cells. Mind that the distal processes of the accessory sheath cells are not depicted in (*A*) but in (*B*) and (*C*). (*B*) Cross-section through the mid-region of the sensillum shaft showing numerous wall pores. The shaft wall cuticle is perforated, giving it a double-walled appearance. (*C*) Cross-section through the basal region of the sensillum shaft which lacks wall pores. Section levels for (*B*) and (*C*) are indicated by lines in *A*. (*D*) Schematic representation of a wp-sensillum featuring the most important similarities and differences compared with (*E*) insect wp-sensilla. (*E*) Representative wall-pore sensillum of insects adopted from Wehner & Gehring: Zoology, Thieme 2013. Labels: *asc* accessory sheath cells, *cu* leg cuticle, *de* dendritic processes, *ds* dendritic sheath, *ecm* extracellular matrix, *epc* epidermal cell, *gc* glial cell, *isc* inner (glandular) sheath cell, *isl* inner sensillum lymph space, *lca* longitudinal shaft wall canals, *msc* median sheath cell, *osc* outer sheath cell, *osl* outer sensillum lymph space, *pasc* digitiform distal processes of accessory sheath cells, *rc* chemoreceptive cell, *sca* spoke canals, *so* socket, *swc* shaft wall cuticle, *wp* wall pores.

Using transmission electron microscopy (TEM), we scrutinized the anatomy of the putative wp-sensilla on the tibia and femur of the first walking leg of male *A. bruennichi*. We hypothesized that these sensilla are olfactory wp-sensilla if 1) the invaginations are indeed pores, 2) the pores lead to a central lymph space in the sensillum shaft, and 3) dendrites are present in the lymph space of the sensillum shaft. In fact, the invaginations continue as pores that traverse the cuticle to a lymph space inside the sensillum (Fig. 1*C*). The cuticle of the shaft wall exhibits canal-like lymph spaces that run longitudinally through the shaft wall (Figs. 1 *C* and *D* and 2 *A*–*C*), similar to double-walled wp-sensilla previously described in sensilla of mites, ticks (14), and harvestmen (15). The double-walled structure is interrupted by spoke canals that connect the outside of the sensillum with the lymph space in the center of the sensillum (Figs. 1*C* and 2*B*). The central lymph space accommodates a varying number of cytoplasmic processes of accessory sheath cells that can be mistaken for dendrites (*pasc* in Fig. 1 *C*–*E*) and hosts a separate compartment with dendrites (Figs. 1 *D* and *E* and 2). The compartment is built by a so-called dendritic sheath that separates an inner from an outer sensillum lymph space inside the shaft (Figs. 1 *C*–*E* and 2). The dendritic sheath runs up to the tip of the sensillum shaft and is produced by the innermost sheath cell (Fig. 2 *A* and *D*). The dendrites inside the inner lymph space are extensions of up to four neurons whose somata are located below the socket region of the sensillum. Cross-sections taken from various levels of these sensilla show that the dendrites and sensillum lymph spaces run all the way up to the sensillum tip. The three characteristics—pores along the shaft wall that connect to lymph space containing dendrites—qualify the sensilla as wp-sensilla. They appear similar to those known from many insects, myriapods, and other arachnids (1, 2, 14, 16, 17). However, the wp-sensilla of the majority of insects are generally devoid of a socket region and a dendritic sheath (Fig. 2*E* and refs. 2 and 16), but, e.g., in carrion beetles wp-sensilla with a continuous dendritic sheath have also been found, and are known to function likewise as olfactory sensilla (18, 19). In arachnids other than spiders, sensilla with wall pores have been described for a few taxa, that seem to differ from the wp-sensilla we found in *A. bruennichi*. For example in ticks (20), ricinuleid spiders (17), and harvestmen (15), the sensilla lack a distinct socket and a continuous dendritic sheath. The whip spider *Heterophrynus* spp possesses wall-pored rod sensilla with a dendritic sheath that does not run all the way up to the tip of the sensillum (figures 23 to 25 in ref. 21) as is also the case for the wall-pore sensillum of the spider *Gradungula sorenseni* (3). Furthermore, some species possess wall-pore sensilla with a single wall (3, 17, 21) or both single- and double-walled sensilla (15, 20). This morphological diversity within arachnids requires further investigation.

The wp-sensilla of *A. bruennichi* males possess sockets, thus their shafts can be slightly deflected, suggesting a mechanoreceptive function. We scrutinized the socket region for integral components of mechanoreceptors of terrestrial arthropods, the tubular bodies (22, 23), and found no tubular bodies. This strongly suggests that wp-sensilla in *A. bruennichi* are unimodal and exclusively serve for chemosensing, similar to the olfactory sensilla of insects (24, 25). This setup is in contrast to the tp-sensilla of *A. bruennichi* males and females, which in fact have a dual function—chemoreception (gustation) and mechanoreception (13, 26, 27).

Taken together, the wp-sensilla of the males of *A. bruennichi* are characterized by a combination of specific features: the socketed sensillum shaft, the double-walled appearance of the shaft cuticle with longitudinal lymph lumina and spoke canals, the extensions of the basal sheath cells into the outer lymph space in the shaft, and the dendritic sheath that surrounds the dendrites up to the tip of the sensillum. This combination of features is not known from any other type of wp-sensillum in arthropods. Structurally, the wp-sensilla of *A. bruennichi* are most similar to the very differently shaped peg-like sensilla basiconica on the antennae of lithobiomorph centipedes (14, 28) and polydesmid millipedes (29), for which a chemoreceptive function has been demonstrated (30). Our ultrastructural examination of several segments of the walking legs of *A. bruennichi* females did not reveal sensilla with wall pores. This is consistent with the results of our FE-SEM analyses where no candidates for wp-sensilla were detected in females and subadult males (SI Appendix, Fig. S5). We conclude that wp-sensilla exclusively occur in *A. bruennichi* males and develop during their last molting stage. In comparison, in insects both males and females possess wp-sensilla, and they can differ in the number or types of sensilla present (2, 31).

## Distribution of Wall-Pore Sensilla in Males

**Fig. 3.**
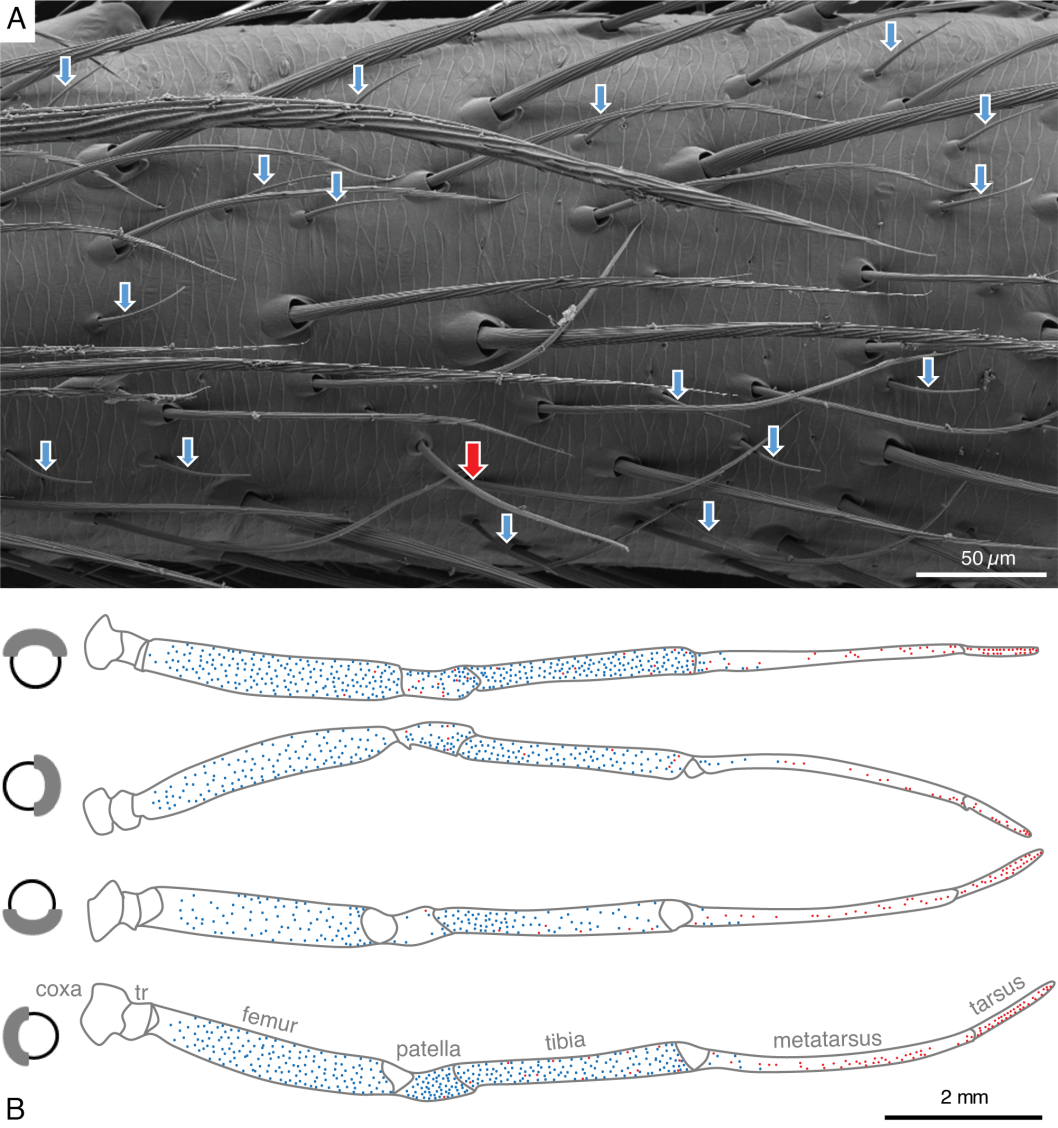
Distribution of wall-pore sensilla (wp-sensilla) and tip-pore sensilla (tp-sensilla) on the first walking leg of an *A. bruennichi* male. (*A*) Representative area of the tibia showing 16 wp-sensilla (light blue arrows) and one s-shaped and longer tp-sensillum (red arrow). (*B*) Distribution map of chemosensilla, viewed from dorsal, prolateral, ventral, and retrolateral (*Top* to *Bottom*). Labels: light blue dots: wp-sensilla, red dots: tp-sensilla; tr: trochanter. See supplement for distribution maps of 2nd, 3rd, and 4th walking legs (SI Appendix, Fig. S2).

In the males of *A. bruennichi*, the wp-sensilla are numerous on the proximal segments of all walking legs, i.e., femur, patella, and tibia. On the more distal metatarsus, there are only a few wp-sensilla (n = 20 to 30). On the tarsus, the most distal podomere, wp-sensilla are absent, while the tp-sensilla are abundant. The distribution of both types of sensilla on the legs is almost complementary with most tp-sensilla on distal leg segments and most wp-sensilla on proximal segments (Fig. 3 and SI Appendix, Figs. S2 and S6). The wp-sensilla are densely distributed over the dorsal, prolateral, and retrolateral sides of the proximal leg segments. A smaller number is found on the ventral side. There are approximately 1,000 wp-sensilla each on the 1st, 2nd, and 4th legs, while the 3rd leg, which is much shorter, exhibits about 450 wp-sensilla (SI Appendix, Fig. S6). A male possesses a total of about 7,000 wp-sensilla. Despite the high abundance of wp-sensilla in *A. bruennichi* males, the pores on the sensilla may have been overlooked in previous studies, as we did when using conventional SEM (5). The resolution of field emission SEM is higher, and a thinner layer of sputter coating is applied. In addition, our previous TEM work (13) focused on the tp-sensilla of the tarsus. In this region, wp-sensilla do not occur in males and therefore could not be sectioned by chance.

## Wall-Pore Sensilla of Males Respond to the Female Sex Pheromone

### Pheromone Assay.

**Fig. 4.**
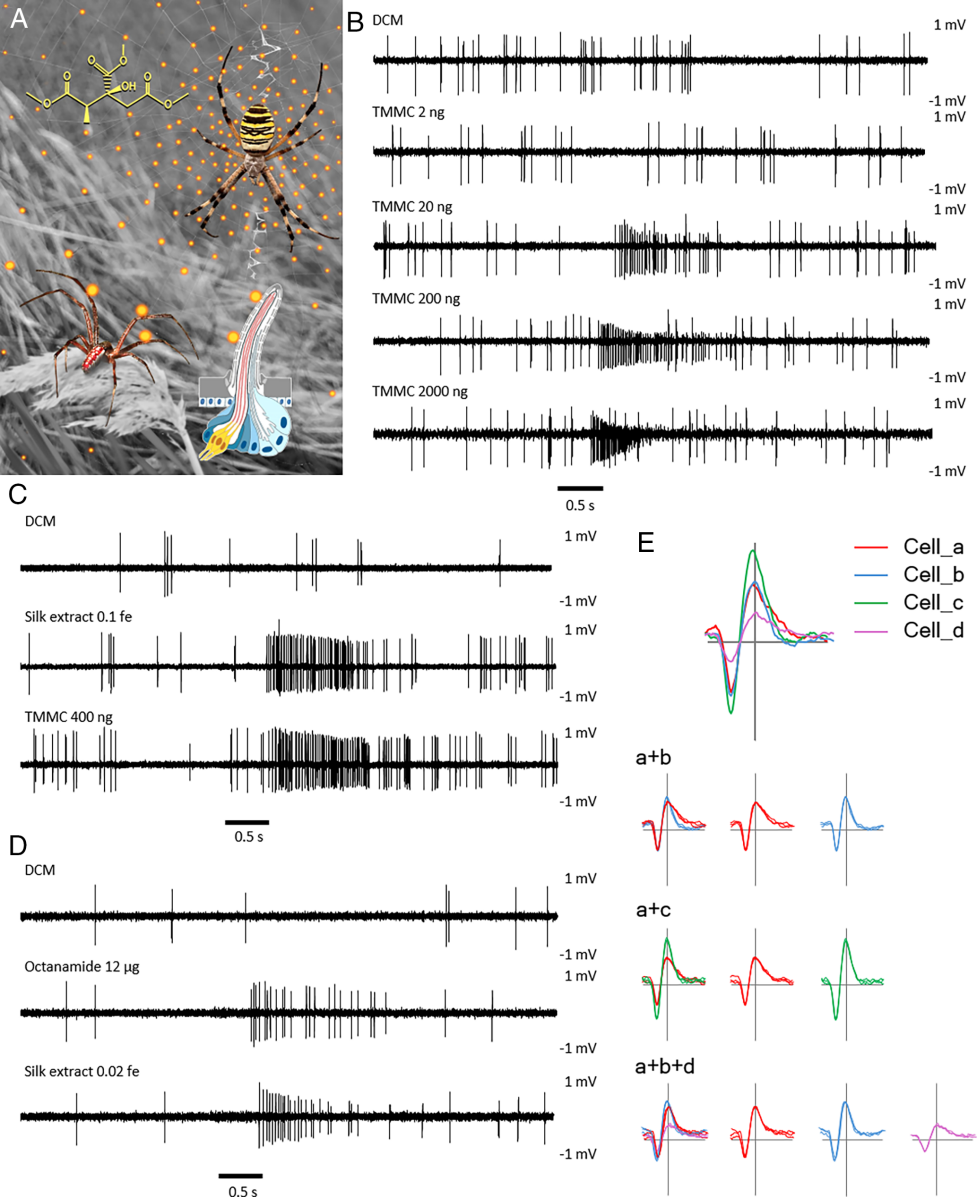
Mate attraction via volatile odor and electrophysiological response of single sensillum recording. (*A*) Scenario of female-produced volatile TMMC captured by wall-pore sensilla of the male *A. bruennichi*. (*B*) Responses of a wall-pore sensillum (femur of the first walking leg of a male *A. bruennichi*) to the synthetic pheromone. TMMC was dissolved in dichloromethane (DCM) and loaded as stimulus to the filter paper in the test tube. Responses to different dosages are labeled in each panel. Bar at bottom shows the 0.5 s puffing period after initial recording of the spontaneous potentials for 2 s. The amplitudes of the potentials are in mV. (*C*) Responses of a wall-pore sensillum (tibia of the first walking leg of a male *A. bruennichi*) to a DCM solvent control, a silk extract of 0.1 female equivalent (fe) that contained about 490 ng of the pheromone, and the synthetic pheromone TMMC in a loading dose of 400 ng. (*D*) Responses of a wall-pore sensillum (tibia of the first walking leg of a male *A. bruennichi*) to a DCM solvent control, a female-silk-contained minor component octanamide in a loading dose of 12 μg, and a silk extract of 0.02 female equivalent (fe) that contained approximately 100 ng of the pheromone. (*E*) Representative pattern of spontaneous action potentials recorded from cohosted neurons in the tested wp-sensilla of *A. bruennichi* males. In total, four physiologically active neurons, named as “Cell_a” to “Cell_d” were observed. These neurons are organized in a binary or ternary cohosting pattern, i.e., a+b, a+c, and a+b+d. The neurons are presented in overlays or separated forms along with the color coding. The pheromone-mediated action potentials were recorded from the neuron Cell_a labeled in red.

In order to test the bioactivity of the synthetically produced *A. bruennichi* pheromone (trimethyl (2*S*,3*R*)-methylcitrate, further called TMMC), we performed bioassays in a seminatural testing arena (*Methods*). Males that matured in the laboratory were released into an arena in the field that contained a pheromone source and a solvent control (loading volume 10 µl). Various concentrations of the pheromone were tested: 2.5 µg/µL, 0.2 µg/µL, 0.1 µg/µL, 0.05 µg/µL, and 0.025 µg/µL. When the male approached and contacted the pheromone source this was recorded as attraction to the pheromone. A concentration of 2.5 µg/µL resulted in 93% attraction probability (N = 15 trials), 0.2 µg/µL in 73% (N = 15), 0.1 µg/µL in 90% (N = 10) and 0.05 µg/µL resulted in 50% (N = 10) attraction probability. The lowest concentration (0.025 µg/µL) was the least attractive (10%, N = 10). The assay demonstrates that the synthetic pheromone attracted males and therefore could be used as a reliable stimulus for single sensillum recording (Fig. 4 and SI Appendix, Fig. S7). The declining attraction probability with decreasing pheromone concentration further suggested concentration-dependent effects.

### Electrophysiology.

We used single sensillum recording to test for an electrophysiological response of the wp-sensilla to the sex pheromone (TMMC) of *A. bruennichi*. In total, 79 wp-sensilla from 26 adult males were tested. The loading of 20 ng of synthetic pheromone as a stimulus was sufficient to elicit clear action potentials (Fig. 4*B*). When the loading amount was increased to 200 ng and 2 µg, stronger responses were observed, indicated by an increased firing frequency (Fig. 4*B*). Under a higher dose, the action potentials from the responding neuron showed gradually reduced amplitudes or, in some cases, the neuron adapted for a few seconds followed by a quick recovery (Movie S2). These results demonstrate that the olfactory neurons of the wp-sensilla are highly sensitive and finely tuned to the sex pheromone of female conspecifics. The response of the wp-sensilla to the pheromone was consistent regardless of which segment of the walking leg or which leg pair of the male was tested.

To compare the response obtained with the synthetic pheromone to the response toward the native pheromone, the crude extract of the web of an adult female *A. bruennichi* was used as stimulus. GC/MS analysis suggested a total of approximately 4.9 µg pheromone in the crude extract (SI Appendix, Fig. S8). When this silk extract was used as stimulus in a dose of 0.1 or 0.02 female equivalents, strong responses were elicited from the same neuron that responded to the synthetic pheromone (Fig. 4 *C* and *D*).

The sensitivity of the olfactory sensory cells in the response of *A. bruennichi* males to the female sex pheromone is impressively high, comparable to the most sensitive pheromone-mediated sexual communication systems found in insects (32–34). In light of the densely distributed wp-sensilla on all walking legs, it seems very likely that *A. bruennichi* males can perceive highly diluted airborne volatiles. In addition, we also challenged tp-sensilla in the nontouch areas by tip-recording, but no convincing response to the pheromone compound was observed in both male and female spiders.

### Other Potential Stimuli of the Wall-Pore Sensilla Neurons.

The wp-sensilla of *A. bruennichi* contain 1 to 4 dendrites based on our ultrastructural findings and judging from the different spike pattern of the spontaneous potentials. However, only one of the neurons, namely “Cell_a” (Fig. 4*E*) responded to the synthetic or natural pheromone in our trials. For two-neuron-hosting sensilla, the spontaneous potentials of the two cells had similar or different amplitudes. In the case of three cohosted neurons, two cells generated relatively larger amplitudes including the pheromone-responding Cell_a (Fig. 4*E*). To explore the response profiles of the other neurons in the wp-sensilla, we tested further compounds as stimuli that were identified from the cuticle and silk extracts of female *A. bruennichi*. These were i) the two enantiomers of the volatile 3-octanoyloxy-4-butanolide (referred to as compound B in ref. 8), ii) tetradecyl (2*S*,4*S*)-2,4-dimethylheptadecanoate, a representative, nonvolatile cuticle wax ester that contains the *Argiope* characteristic 2,4-dimethyl group (35), and iii) octanamide, a minor component that we identified in the female silk extract that contains an octanoyl character structure as does the abovementioned lactone (SI Appendix, Fig. S8). We also tested iv) the degraded products of the cuticle wax ester (SI Appendix, Fig. S9) that contained 2,4-dimethylheptadecanoate in the form of its methyl ester and tetradecanol. Different amounts of these compounds, ranging from 200 ng to 20 µg per loading, were applied in single sensillum recordings. Except for octanamide, which elicited a relatively weak response from the pheromone-responsive neuron under higher loading dose (Fig. 4*D*), the other compounds did not elicit responses in the pheromone-responsive neuron, nor in other neurons of the wp-sensilla. In conclusion, we found that one olfactory receptor neuron in the wp-sensilla of *A. bruennichi* always reacted to the sex pheromone. The roles of the additional neurons in the wp-sensilla remain to be explored. Possibly, nontargeted metabolomics together with high-performance liquid chromatography-mass spectrometry (HPLC-MS) and GC-MS (36) might help to discover pheromone candidates that were not detected with the targeted approaches performed so far (8, 35). Another possibility is that the neurons respond to some pheromone components of closely related and sympatric spider species. Such active ligands can serve as antagonists to avoid attraction to interspecific females as found in studies on insects (37–39). In the Mediterranean area, the core area of *A. bruennichi* in Europe, several species of *Argiope* can co-occur on the same meadow (*A. bruennichi*, *A. lobata,* and *A. trifasciata;* Uhl personal observation) and preliminary data suggest that trimethyl methylcitrate is broadly used as a sex pheromone in *Argiope* species (40), detailed stereochemistry unknown. Interspecific attraction might have fatal consequences due to the pronounced sexual cannibalism in *Argiope* species (11) unless other species-specific components come into play. The minor but active octanamide that we found in the silk extract of *A. bruennichi* females is an example of an active compound that was missing or was overlooked in previous studies (8, 40).

## Beyond *A. bruennichi*: A Comparison of Wall-Pore Sensilla across the Spider Tree of Life

**Fig. 5.**
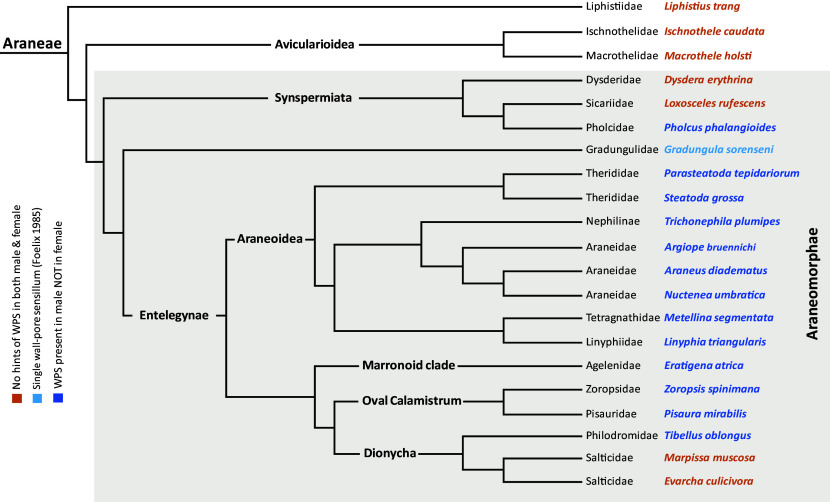
Taxon sampling for FE-SEM-based screening for wall-pore sensilla in the spider (Araneae) tree of life. Adapted from refs. 44–46.

We examined male and female specimens from 19 additional species of 16 families across the spider tree of life for the presence of wp-sensilla (Fig. 5). Using FE-SEM, we scrutinized the retrolateral side of the first walking leg of male and female spiders. Trichoid sensilla with pore-like depressions on the sensillum shaft wall were considered putative wp-sensilla. Sensilla with putative wall pores were observed in male individuals in all seven examined species of the Araneoidea superfamily. These include two additional araneids, *Araneus diadematus* (Araneidae) and *Nuctenea umbratica* (Araneidae), as well as *Trichonephila plumipes* (Nephilinae), *Metellina segmentata* (Tetragnathidae), *Linyphia triangularis* (Linyphiidae), *Parasteatoda tepidariorum* (Theridiidae), and *Steatoda grossa* (Theridiidae) (SI Appendix, Figs. S10–S12). In taxa from the Marronoid clade (*Eratigena atrica*) and Oval Calamistrum clade (*Pisaura mirabilis, Zoropsis spinimana*) we found putative wp-sensilla, also only in male specimens (SI Appendix, Fig. S13). Likewise, *Tibellus oblongus* (Philodromidae), a species from the Dionycha clade, possesses putative wp-sensilla and also only in males (SI Appendix, Fig. S14 *A*–D). The suggested exclusive occurrence of wp-sensilla in adult males supports the use of these sensilla for sex pheromone detection and possibly other substances that play a role during mating as outlined above. However, the lack of wp-sensilla in females and subadult spiders also raises the question of whether they cannot perform olfaction, or if they do so via different sensilla. One possibility is that tp-sensilla—which are abundant in all stages and both sexes of all studied spiders—serve not only for gustation but also for olfaction. Literature supports the idea that olfaction is possible via tip-pore sensilla (41), but electrophysiological tests on tp-sensilla in spiders have so far only been conducted for contact chemoreception (4). Another possibility is that living in a web has relaxed the need for olfaction in females since the presence of prey and predators can be detected by the web, which serves as an extended phenotype. However, this argument cannot explain the lack of wp-sensilla in female wandering spiders such as *P. mirabilis* and *T. oblongus,* nor the complete lack of wp-sensilla in salticids such as *Marpissa muscosa* and *Evarcha culicivora* (both Salticidae) (SI Appendix, Fig. S15). For salticids, behavioral data on mate-odor detection are available (42), which is why we chose to scrutinize *E. culicivora*. In this species males, females, and juveniles can identify mosquitoes that have taken a blood meal by odor alone (43). Possibly, salticids perform olfaction either using tp-sensilla or with as yet undiscovered structures.

The occurrence of wp-sensilla in the inspected 20 species is illustrated in Fig. 5. The most basally branching spider lineages—*Macrothele holsti* (Macrothelidae), *Ischnothele caudata* (Ischnothelidae), and *Liphistius trang* (Liphistiidae)—lack wp-sensilla in both males and females (SI Appendix, Fig. S17), strongly suggesting that wp-sensilla evolved within Araneae. Interestingly, within the clade of Synspermiata, male *Pholcus phalangioides* (Pholcidae) possess putative wp-sensilla (SI Appendix, Fig. S14 *E*–H), while in *Dysdera erythrina* (Dysderidae) and *Loxosceles rufescens* (Sicariidae) no putative wp-sensilla were detected (SI Appendix, Fig. S16), suggesting convergent evolution or loss events. Fig. 5 also includes the finding by Foelix (3) of a wp-sensillum on the tarsus in the spider* G. sorenseni* (Gradungulidae) endemic to Australia and New Zealand. Interestingly, the sensillum differs in ultrastructure from the wp-sensilla of *A. bruennichi* in that there is a single, strongly branched dendrite making the wp-sensillum of *G. sorenseni* more similar to those found in whip spiders (3, 21). However, the sex of the specimen that was inspected is not known, and whether there is more than one sensillum remains to be investigated.

Although our taxon sampling is admittedly preliminary and the categorization of the presence and absence of wp-sensilla is based on SEM inspection only, the comparative data allow formulating hypotheses about the evolution of wp-sensilla in spiders, as well as arachnids and arthropods. For spiders (Araneae), the current data suggest three occurrences of convergent evolution of wp-sensilla: one within the Synspermiata (i.e., Pholcidae), one at the base of the Gradungulids, and one at the base of the Entelegynae. Since we did not detect wp-sensilla in salticids, we hypothesize that they were lost at the base of the salticids.

Within arachnids other than spiders, multiporous sensilla with single or double walls have been identified in various taxa. Examples include whip spiders [Amblypygi (3)], harvestmen [Opiliones (15)], ticks, mites [Acari (47)], and hooded-tick spiders [Ricinulei (17, 48)]. These sensilla vary considerably in ultrastructural anatomy, suggesting multiple origins of wp-sensilla in these arachnid groups. Hallberg and Hansson (49) noted that the evolutionary pathway of olfactory sensilla in insects is difficult to trace, not least because of the enormous diversity of morphotypes found in different lineages, and they surmised that cuticular sensilla are prone to convergence due to their relatively simple anatomy and constraints imposed by functional morphology. This statement might be extended to arachnids.

In summary, wall-pore sensilla are present in adult *A. bruennichi* males on all walking legs and respond to the pheromone produced by females. Their anatomical features differ in several respects from those known from insects. Assuming that our morphological and physiological findings on *A. bruennichi* can be applied to other entelegyne spiders with wp-sensilla, the abundance of thousands of sensilla and their sensitivity toward the sex pheromone strongly suggest that wp-sensilla in male spiders are finely tuned to the pheromone to find the female during the often short reproductive period. The finding that only one neuron out of a maximum of four neurons in the wp-sensilla of *A. bruennichi* males responded to the female pheromone suggests further, as yet undetected chemosensory functions of these wp-sensilla, probably related to species discrimination, mate detection, and mate assessment.

Our comparative study, although preliminary, suggests multiple origins of wp-sensilla and even the loss of wp-sensilla within the Araneae. Since we found no candidates for wp-sensilla in the basally branching Araneae, olfactory sensilla probably evolved within the spiders and also—based on the limited evidence available to date—probably several times convergently in arachnid groups such as the opilionids, amblypygids, and ticks.

## Methods

### Spider Collecting and Maintenance.

Subadult and adult males and females of *A. bruennichi* (Scopoli, 1772) were collected in the summers of 2021, 2022, and 2023 from various locations in northern Germany. They were then reared in a temperature-controlled climate chamber at a temperature between 22 °C and 25 °C. Subadult males were housed individually in cylindrical plastic containers (10 cm high, 5 cm diameter). The containers were fitted with two strips of adhesive tape to allow the subadult males to build a web. A cotton pad was moistened every second day which was fixed between the wall of the container and a foam stopper. Subadult and adult males were fed with fruit flies (*Drosophila hydei*) twice a week. Subadult and adult females were kept in larger plastic containers (13 cm height, 12 cm diameter). They were fed with house flies (*Musca domestica*) three times a week and sprayed with water every second day.

### Behavioral Analysis.

#### Contact and noncontact areas of body appendages.

To delineate the contact and noncontact areas on body appendages of male and female *A. bruennichi*, we observed six adult males and six adult females in different contexts. The animals were carefully transferred from their container to substrates that they might encounter in their natural habitat. Each spider was placed on the following substrates: a flat surface, a horizontal artificial leaf, a horizontally oriented skewer (20 cm long) (Movie S1), and a bundle of natural grass (about 30 cm long). Spiders were also placed on webs built by females in a perspex frame (35 cm × 35 cm × 12 cm). We also observed contact between males and females during mating (N = 6 pairs). We placed a virgin female spider in a plastic frame (dimensions as above), where she built an orb-web and remained in the typical position and posture—facing downward in the hub of the web. Every female was paired with a single inexperienced male. The male was released in the periphery of the web and typically quickly approached the female in the hub, then walked around the female touching her legs and opisthosoma, using mainly his first and second pairs of legs. The male produced vibrations by jerking in the web and bobbing with his opisthosoma. Receptive *A. bruennichi* females raise their opisthosoma to a horizontal position and lift the scape, a shoehorn structure that covers the genital openings, thereby facilitating mating.

We recorded the movements of males and females on the substrates and during mating using a high-speed camera (Miro LC320S, AMETEK Vision Research) at 500 frames per second. For each trial, new grass and freshly built webs were used, and surface substrates were cleaned with 70% ethanol and air-dried after each trial. We analyzed the video clips in slow motion using Image J software (public domain, https://imagej.net/ij/). We recorded which segments (podomeres) of the body appendages did not make contact in the different contexts and which did. Podomeres that touched the substrates at least once during a trial were categorized as contact areas for the specific leg in that trial. The number of trials in which contact was observed was related to the total number of trials (all substrates and mating partner), resulting in a range of 0 to 100%. Podomeres that contacted the substrate or mating partner in more than 50% of cases of were designated as frequent contact areas, while those below 50% were considered infrequent contact areas. Frequent and infrequent contact areas are color coded in SI Appendix, Fig. S1 and data are given in SI Appendix, Table S1. In *A. bruennichi*, only adult females produce a web with which they capture prey. Therefore, we performed additional observations on contact and non-contact areas during prey capture for females only. *Lucilia caesar* flies were used as prey and the recordings were done as described above and data are given in SI Appendix, Table S1.

#### Synthetic sex pheromone: Testing in a seminatural setup.

The sex pheromone of *A. bruennichi* - (*2R*,*3S*)-trimethyl methylcitrate (TMMC) - was synthesized by ASM research chemicals, Hanover, Germany, based on the protocol described in ref. 8. To test the bioactivity of the synthetic pheromone before starting the electrophysiological experiments, we performed a seminatural field test on the premises of Lund University over three consecutive days in mid-July 2023. The synthetic pheromone was dissolved in dichloromethane at different concentrations (2.5 µg/µL, 0.2 µg/µL, 0.1 µg/µL, 0.05 µg/µL, 0.025 µg/µL). We coated wooden skewers at one end with paraffin (Sigma Aldrich) to prevent rapid evaporation of the synthetic pheromone. Perspex frames measuring 29 cm × 29 cm (height 9 cm) were placed on a meadow. Immediately before the start of a trial, 10 µL of a pheromone solution of specific concentration was added carefully to the paraffin head of a skewer using a pipette. Another skewer was prepared right before or after (in random order) as a solvent control by applying 10 µL of the solvent (dichloromethane). All five pheromone solutions were tested on all days in a near-random order. One “trap” containing TMMC and a control skewer were placed in two corners of the frame (29 cm apart). Immediately afterward, males that had matured in the laboratory were carefully released opposite the trap and control at the same distance from both. The behavior of the males was observed for 15 min. For each of the above concentration, 10 to 15 trials were conducted. Males were used only once.

We classified males as successfully attracted when they approached and climbed on the skewer, exhibited bobbing courtship behavior, and then remained in the vicinity of the skewer and deposited silk in the nearby vegetation (up to 6 cm from the skewer). After each trial, the frames were cleaned with 70% ethanol, air-dried, and then moved to a new location. The positions of the skewers with the pheromone and controls were randomly changed between trials.

### Anatomy.

#### Scanning electron microscopy: Examination and mapping of sensilla.

Freshly molted adult males and females of *A. bruennichi* were anesthetized with CO_2_ and then mounted on a polyethylene board (Plastazote) with their legs stretched out, fixed with wire clamps and preserved in 80% ethanol. The walking legs and pedipalps of the left body side were then separated from the body and dehydrated in an ascending series to 99% ethanol and then critical point dried using a Leica EM CPD300. The straightened legs and the pedipalps were mounted horizontally on double-sided copper tape on metal SEM stubs using conductive silver (Silver DAG—4-Methylpentan-2-on, ACHESON) to maintain electrical conductivity. Since the copper tape was longer than the stub, this allowed bending the sample at various angles. In order to obtain an overview of the sensilla distribution, the samples were coated with gold-palladium for 150 s using a Polaron SC7640 sputter coater (Fisons Instruments) and analyzed under an SEM (EVO LS 10, Zeiss, Germany) with 10 kV. The body appendages were oriented perpendicular to the electron beam and were rotated 0°, 90°, 180°, and 270° around the long axis of the appendage by bending the copper tape. Accordingly, images were taken from dorsal, prolateral, ventral, and retrolateral sides of the body appendages. All segments (podomeres) of the appendages (coxa to tarsus) were imaged at a constant magnification in the SEM. The images were carefully stitched together at the overlap regions, and the relative positions of the focal sensilla were reconstructed on outlines of the spider’s legs. These outlines of legs were derived from multifocus stereomicroscopic photographs (Zeiss SteREO Discovery.V20) that captured all four perspectives (dorsal, prolateral, ventral, and retrolateral).

A field emission SEM (SUPRA 40VP, Zeiss, Germany) was used to inspect the surface of the sensilla with high resolution. Males, females, and subadult males were investigated. The surface of the samples was cleaned with a 5% KOH solution and glacial acetic acid (50). Critical point-dried samples were mounted on SEM stubs and sputter-coated with an AU-Pd (80:20) mixture at 5 mA (Q150T ES, QUORUM, UK). The thickness of the coating was 10 nm.

As we found wall-pore sensilla in the males of *A. bruennichi*, we made a comprehensive map of the sensilla distribution for one individual. Additional males were examined and individual variation was found to be low. When counting the total number of wall-pore sensilla based on the maps, we excluded all wall-pore sensilla that appeared on several maps from different perspectives due to overlap. All measurements of the different parameters of the wall-pore sensilla of males (length, diameter) were conducted using the built-in software of the FE-SEM.

#### TEM: Ultrastructural examination of sensilla.

Four males and two females of *A. bruennichi* were anesthetized using CO_2_. Subsequently, all walking legs were carefully dissected with microscissors in a cold prefixative solution modified after Karnovsky’s protocol (51) (2.0 % glutaraldehyde, 2.5% paraformaldehyde, and 1.5% sucrose solved in a 0.1 mol/L sodium phosphate buffer at a pH of 7.4). To enhance the quality of tissue preservation, each podomere was cut into two to five pieces, of approximately 1 to 3 mm length, and incubated in the same prefixative solution. To further enhance fixation quality, the samples were subjected to three sets of 2-min pulses of microwave radiation, operating at a power of 120 W, using a PELCO BioWave Pro BioWave equipped with the PELCO SteadyTemp Pro Thermo Cube for solid-state cooling. Throughout the BioWave application, the sample temperature was carefully monitored and maintained between 8 °C to 10 °C before and 14 °C to 19 °C after treatment. Samples were then stored in the same prefixative solution at 4 °C at least overnight. To maximize fixation quality and contrast of the target tissue, we modified an existing protocol (52). Samples were washed four times in 0.1 M sodium phosphate buffer over a period of 2.5 h at 4 °C. After washing, the samples were postfixed with 2% osmium tetroxide (dissolved in the same buffer) for 12 to 20 h at 4 °C. The samples were then rinsed for 2 h at 4 °C in a buffer solution containing potassium ferrocyanide (1% FeCN in 0.1 M SB). They were then washed four times with double-distilled water (ddH2O,) for a total of 2.5 h at 4 °C. The samples were then stained en bloc with a heated (60 °C) 1% aqueous uranyl acetate solution (solved in ddH_2_O) at 4 °C for 8 to 12 h. After staining, the samples were washed four times with ddH_2_O over a period of 2 h at room temperature. The staining was enhanced by further treatment with lead aspartate (0.66% lead nitrate solved in 0.03 M aspartic acid, adjusted to a pH of 5.5) for 2 h at 50 °C. The samples were then washed four times with ddH_2_O, for a total of 2.5 h at room temperature. The samples were dehydrated with a series of ethanol (50%, 60%, 70%, 80%, 90%, and 100%). Up to the 90% ethanol stage dehydration was done for 10 min at room temperature. For the 100% ethanol stage, the samples were washed three times for 10 min each at room temperature. Subsequent stepwise infiltration of the samples with epoxy resin Embed812 (Epon substitute) was performed at room temperature using propylene oxide (PO) as solvent (2× 100% PO for 15 min, 66% for 2 h, 50% for 12 h, and 33% for 24 h), with the samples constantly moved on a shaker. For pre-embedding, the samples were placed in 100% epoxy resin on a shaker at room temperature for 2 h. Then the samples, still in 100% epoxy resin, were placed in a Heraeus VT-6025 vacuum heating cabinet for a further 2 h, operated at 150 mbar and 40 °C (three times 30 min with 10 min break in between under normal atmospheric pressure). For final embedding, the samples were transferred to silicon embedding molds containing new epoxy resin. Each piece of the femur, trochanter, tibia, metatarsus, and tarsus was individually oriented in a specific resin block (transverse, oblique, or longitudinal orientation to the block face). The resin blocks were polymerized in a heating cabinet at 60 °C for 48 h.

Transverse, horizontal, and longitudinal ultrathin sections (70 to 90 nm in thickness) of the tibia and femur were produced using a LEICA UCT ultramicrotome. In areas from which ultrathin sections were obtained (2 to 5 µm trimming gaps), we also took semithin sections (700 nm thickness), which were stained with toluidine blue (solved in 1% sodium tetraborate) for examination under a light microscope. The ultrathin sections were transferred to Formvar-coated slot grids (PLANO) and examined with a JEOL JEM-1011 TEM at 80 kV. Digital photomicrographs were taken with a mid-mount camera (Mega View III, Soft Imaging System) and the iTEM imaging software (Soft Imaging System).

### Electrophysiology.

#### Single sensillum recording on wall-pore sensilla of males.

The walking legs of adult male spiders were fixed on a microscope slide with dental wax, with the leg positioned so that light could be applied from below (SI Appendix, Fig. S7*A*). The thickest sensilla (likely mechanosensilla) on the podomeres were removed manually with a finely polished wire to gain access to the wall-pore sensilla with the recording electrode. The sample was mounted on a Nikon microscope (Nikon, eclipse E6000FN) equipped with 10× and 50× magnification objectives. A reference tungsten electrode was electrolytically sharpened and inserted into the posterior end of the spider’s opisthosoma. A tungsten recording electrode was inserted into the base of a wall-pore sensillum to contact the olfactory sensory neurons inside (SI Appendix, Fig. S7*B*). A constant flow of charcoal-filtered and humidified air was directed to the podomere that contained the target wp-sensillum at a rate of 1 L/min. The stimulus was loaded onto a piece of filter paper (2 cm^2^, Whatman) inserted into a Pasteur tube. A stimulus controller (Syntech CS-02, Buchenbach, Germany) was used to puff the stimulus in the Pasteur tube into the airstream (0.5 s, 0.3 L/min) through a glass tube (6 mm in diameter), with the outlet 20 mm away from the target sensillum.

The recording electrode was connected to a Sensapex uMp micromanipulation system (Sensapex Oy, Finland) to enable finely controlled movements. A 10× gain DC probe and an IDAX4 controller (Syntech) were used to capture and amplify the signals using Autospike software (Synthetic, V.3.9, Buchenbach, Germany), digitalized via an analog–digital converter and then visualized and made audible by a computer screen and PC speaker, respectively.

Synthetic pheromone (2*R*,3*S*)-trimethyl methylcitrate and other compounds identified from cuticle and silk extracts, including the (*S*-*/R*-) enantiomers 3-octanoyloxy-4-butanolide, cuticle wax ester tetradecyl (2*S*,4*S*)-2,4-dimethylheptadecanoate and corresponding hydrolyzed acid and alcohol, as well as octanamide identified from the female silk extract, were dissolved separately in dichloromethane by desired concentration and loaded on the filter paper. A loading of 10 µL dichloromethane was used as solvent control. The glass tube delivering the stimulus was cleaned every day after use. Pipettes of stimulus were mostly used for a maximum of 3 h.

#### Recording of tip-pore sensilla.

For tip-recording, male and female adult spiders were mounted on a microscope slide the same way as described above. A reference silver electrode was inserted into the posterior end of the opisthosoma of the spider. A glass recording electrode was prepared using a micropipette puller (Model P-87, Sutter Instrument Co.), equipped with a silver wire to conduct electricity, and filled with 0.1 M sodium chloride solution containing 1% methanol, with or without the pheromone compound as stimulus or solvent control, respectively. The pheromone (2*R*,3*S*)-trimethyl methylcitrate was first dissolved in methanol and then diluted in 0.1 M sodium chloride with a final concentration of 124 µg/µL pheromone (equivalent to 0.5 M) and 1% methanol. The recording electrode was finely controlled to successively contact the tip-pore sensilla along the tarsus and metatarsus. The potentials of the sensory neurons were recorded for 30 s at each contact. The data were recorded using Autospike software (Synthetic, V.3.9, Buchenbach, Germany).

### Chemical Analysis.

#### Pheromone extraction.

To obtain native pheromone for comparison, web silk from a virgin adult female, about 3 wk old, was collected using a glass micropipette (Blaubrand, 200 µL) and extracted overnight in 1 mL dichloromethane. The silk extract was then transferred to GC vial and concentrated to 100 µL prior to GC/MS analysis.

#### Gas chromatography and mass spectrometry.

The silk extract was analyzed using an Agilent 5977B mass detector coupled to an Agilent 8890 series gas chromatograph equipped with an HP-INNOWax column (30 m × 0.25 mm i.d., 0.25 μm film thickness; J&W Scientific, Agilent Technologies, Santa Clara, CA). The GC inlet was set to 250 °C and the oven program was set to 80 °C for 1 min, increased by 10 °C/min to 230 °C, then held for 10 min. The temperature for the transfer line and MS source was set to 280 °C and 230 °C, respectively. Helium was used as the carrier gas at a flow rate of 1 mL/min. The pheromone compound (2*R*,3*S*)-trimethyl methylcitrate was identified from the silk extract by comparing the retention times and mass spectra with those of the synthetic compound.

### Chemical Synthesis.

#### (*R*)- and (*S*)-3-octanoyloxy-4-butanolide.

The synthesis of the 3-octanoyloxy-4-butanolide enantiomers was previously described (8).

#### Octanamide.

Octanamide was identified as a minor component in the silk extract of females. A sample of 0.5 g octanoic acid was heated to 40° C and then 0.5 mL thionyl chloride was added under a N_2_ atmosphere over a period of 15 min. The reaction mixture was heated to 110° C for 30 min to obtain crude octanoyl chloride. Then 1.5 mL of conc. ammonia solution was placed in a 50 mL flask and cooled to 10° C. The freshly prepared octanoyl chloride was added at 8 to 12° C for 30 min, the reaction mixture was stirred at 10° to 15° C for 10 min and then stirred at room temperature for 1 h. The organic layer containing crude octanamide was collected and rinsed successively with 1 mL 20% NaCl solution and water and then dried over sodium sulfate. Octanamide was quantified on GC/MS under the same condition as described above.

#### Alcoholysis of the wax ester tetradecyl (2S,4S)-2,4-dimethylheptadecanoate.

The synthesis of the wax ester tetradecyl (2*S*,4*S*)-2,4-dimethylheptadecanoate was described previously (35). To obtain the transesterified products of the ester, 1 mg of (2*S*,4*S*)-2,4-dimethylheptadecanoate was added to a 0.5 M KOH/methanol (1 mL) solution. After 1 h at room temperature, the degraded products, the corresponding methyl dimethylheptadecanoate and tetradecanol, were extracted by heptane and quantified by GC/MS. The organic solvent was then evaporated under a stream of N_2_ and the residues were taken up in dichloromethane for electrophysiological tests.

### Comparative Study.

#### Spider collection and maintenance.

*L. trang* (Liphistiidae) from Trang province, Thailand, as well as *M. holsti* (Macrothelidae) from Taiwan were collected by Niklas Reinhardt. *I. caudata* (Ischnothelidae) were obtained from a laboratory culture of Matthias Pechmann. *Dysdera erythrina* (Dysderidae) were collected in 1999 by Gabriele Uhl in Freiburg, Germany. *T. plumipes* (Nephilinae) were obtained from Jutta Schneider. *L. rufescens* (Sicariidae) and *Z. spinimana* (Zoropsidae) were collected in September 2023 and March 2024 by Carsten Müller on Ibiza, Spain. We collected *P. phalangioides* (Pholcidae) and *N. umbratica* (Araneidae) in September 2023 in Greifswald, Germany*. A. diadematus* (Araneidae) were collected as subadult individuals in Greifswald, Germany, and reared in a temperature-controlled climate chamber until they reached their final molt in August 2023. *M. segmentata* (Tetragnathidae) and a female *L. triangularis* (Linyphiidae) were collected in September 2023 near Greifswald, Germany, by Gabriele Uhl. A male *L. triangularis* was collected in 2015 on Hiddensee, Germany, by Gabriele Uhl. A male *E. atrica* (Agelenidae) was collected in September 2023 and a female was collected in 2012 from Greifswald, Germany, or nearby by Gabriele Uhl. *P. mirabilis* (Pisauridae) were collected as subadults in April 2023 in Greifswald, Germany, and reared in the lab until they reached their final molt in May 2023. *T. oblongus* (Philodromidae) were collected in 2020 in Stralsund, Germany, by Heidi Land. Philip Steinhoff collected *M. muscosa* (Salticidae) from Greifswald, Germany, in 2022. Male and female *P. tepidariorum* (Theridiidae) were obtained in 2023 from lab culture run by Em Steiger. *S. grossa* (Theridiidae) were collected as juveniles and subadults by Andreas Fischer at the Burnaby campus of Simon Fraser University (Burnaby, BC, Canada). They were reared in a climate chamber until they reached their final molt in November 2023. *E. culicivora* (Salticidae) were collected in Mirogi, Kenya, by Robert Jackson, Florida State Collection of Arthropods (#k17/10). All spiders were preserved in 70% ethanol.

#### Scanning electron microscopy: Screening for putative wall-pore sensilla in 19 other spider species.

The first walking legs of males and females of all spider species of the comparative study were investigated. The surfaces of the samples were cleaned with a 5% KOH solution and glacial acetic acid (50). Critical point-dried samples were mounted on SEM stubs and sputter-coated with an AU-Pd (80:20) mixture at 5 mA (Q150T ES, QUORUM, UK). The thickness of the coating was 10 nm. A field emission SEM (SUPRA 40VP, Zeiss, Germany) was used to analyze the surface of sensilla of all studied spider species with high resolution.

## Supplementary Material

Appendix 01 (PDF)

Movie S1. **Contact and non-contact areas on body appendages of a male *A. bruennichi***. Slow-motion footage (500 frame per second) of a male walking on a horizontal skewer. The video highlights that only the distal leg segments make contact with the surface.

Movie S2. **Electrophysiological responses to the female sex pheromone recorded from a wall-pore sensillum on the leg of a male spider *Argiope bruennichi***. The pheromone compound (*2R,3S*)-trimethyl methylcitrate was applied as stimulus on the filter paper in a test tube and puffed into the air flow with different doses, i.e., 20 ng, 200 ng and 2000 ng sequentially at 7.5 s, 22 s and 41 s for 500 ms. The amplitudes of the potentials are given in mV. Three neuron cells (referring to pattern a+b+d in Fig. 4E) were observed based on their spontaneous potential. Under the high dose, the pheromone responding neuron was adapted for a few seconds followed by a quick recovery. A 10x gain DC probe and an IDAX4 controller (Syntech) were used to capture and amplify the signals using Autospike software (Synthetic, V.3.9, Buchenbach, Germany).

## Data Availability

All study data are included in the article and/or supporting information.
