# Supplementary material for: Olfaction with legs—Spiders use wall-pore sensilla for pheromone detection
Source: Proc Natl Acad Sci U S A. 2025 Jan 6;122(3):e2415468121. doi: 10.1073/pnas.2415468121 (PMC11760499; doi:10.1073/pnas.2415468121)
Supplement: Supplementary file 1 — Appendix 01 (PDF) [file pnas.2415468121.sapp.pdf]

Supporting Information for

Olfaction with legs – Spiders use wall-pore sensilla for pheromone detection

Authors: Mohammad Belal Talukder[a], Carsten H.G. Müller[a,1,2], Dan-Dan Zhang[a,b], Stefan Schulz[c], Christer Löfstedt[b], Hong-Lei Wang[b,1,2], Gabriele B. Uhl[a,1,2]

Affiliations:

[a]General and Systematic Zoology, Zoological Institute and Museum, University of Greifswald; Greifswald, 17489, Germany

[b]Pheromone Group, Department of Biology, Lund University; Lund, 22362, Sweden

[c]Chemical Ecology, Institute of Organic Chemistry, Technische Universität Braunschweig; Braunschweig, 38092, Germany

[1]Equal contribution

[2]Corresponding authors. Email: gabriele.uhl@uni-greifswald.de; hong-lei.wang@biol.lu.se; carstmue@uni-greifwald.de;

This PDF file includes:

Supporting text
Figures S1 to S17
Table S1
Legends for Movie S1 and S2

Other supporting materials for this manuscript include the following:

Movie S1 and S2

Supporting text

Body appendages: areas of contact and non-contact with substrates

To investigate which areas on the walking legs and pedipalps of *A. bruennichi* come, or do not come into direct contact with the immediate surrounding, we conducted behavioral experiments using high-speed video recording (Movie S1). We hypothesized that the areas that are never observed in contact would be the most likely candidate areas in our search for olfactory sensilla in spiders. We evaluated which regions of the appendages did and did not come into contact with the substrate in different contexts: while walking on different substrates, while dealing with prey, or while mating (see methods of main text). The most distal segment of male and female walking legs, the tarsus, was always in contact with the substrates (Table S1). The following segment, the metatarsus, was less frequently in contact with the substrate (3–16 % during walking and 16–66% during mating). The other leg segments that follow towards the body (tibia, patella, femur, coxa) did not contact the substrate in all tested contexts (Table S1). As for the pedipalps – paired appendages between chelicerae and walking legs – their tarsi frequently come into contact with the substrate in females (70%), who mainly use the pedipalps to explore the environment and prey by touch. In males, the pedipalps serve as sperm storage and transfer organs. The male pedipalps touched the substrate only occasionally (20%), but always touched the female during mating (Table S1). The proximal segments of male and female pedipalps were never in contact with the substrates. Because adult males do not produce webs and do not capture prey, the comparison of males and females touch and no-touch areas in Fig. S1 is based on the contexts "walking" and "mating" only.

Supporting Figures

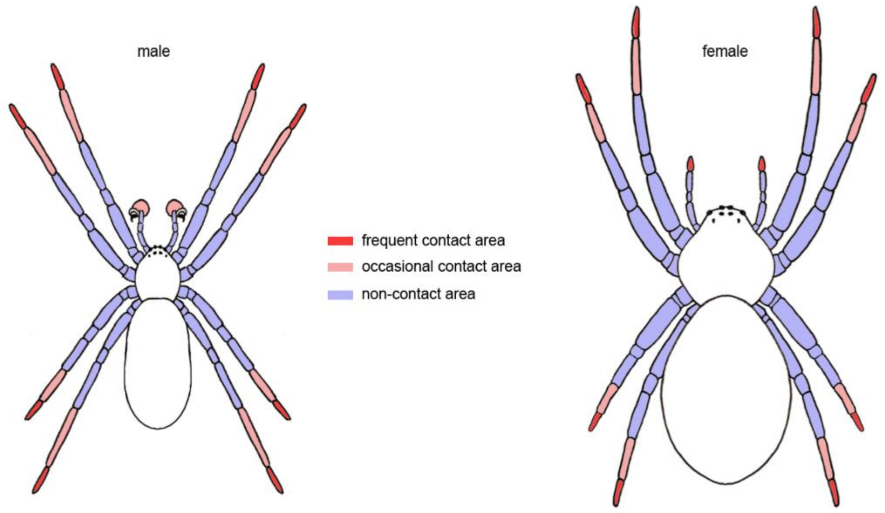

Fig. S1. Contact and non-contact areas of the walking legs and pedipalps in *A. bruennichi* males and females established by high-speed video recording. Since males are not producing webs and do not capture prey this comparison is based on data from the contexts “walking” and “mating”. Segments from tip to body: tarsus, metatarsus, tibia, patella, femur, trochanter, coxa.

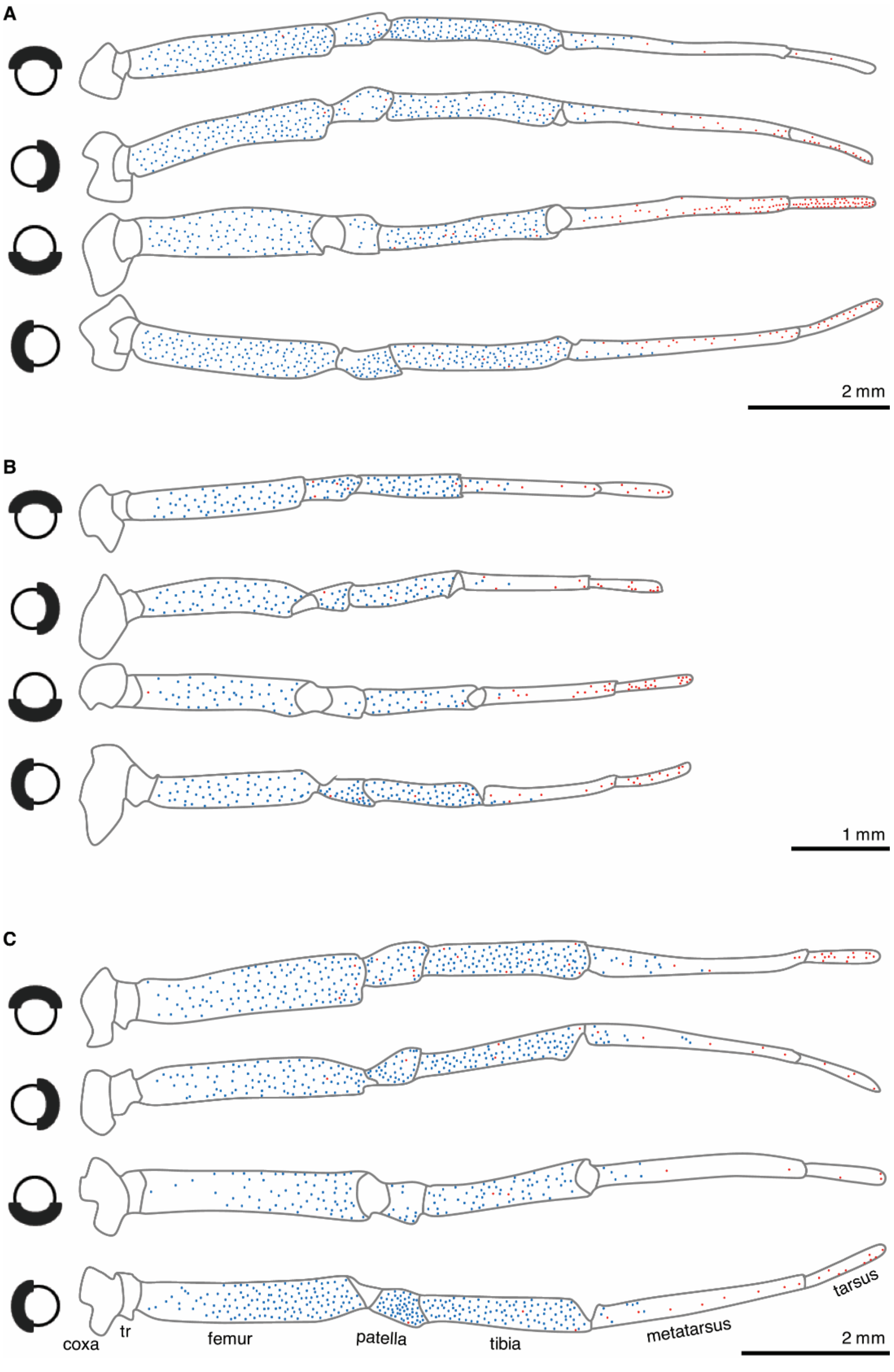

Fig. S2. Distribution of wall-pore sensilla and tip-pore sensilla on the walking legs of male *Argiope bruennichi*. (**A**) 2nd walking leg. (**B**) 3rd walking leg. (**C**) 4th walking leg. Light blue dots represent wp-sensilla and red dots represent tp-sensilla. Top to bottom: dorsal, prolateral, ventral and retrolateral view. tr: trochanter.

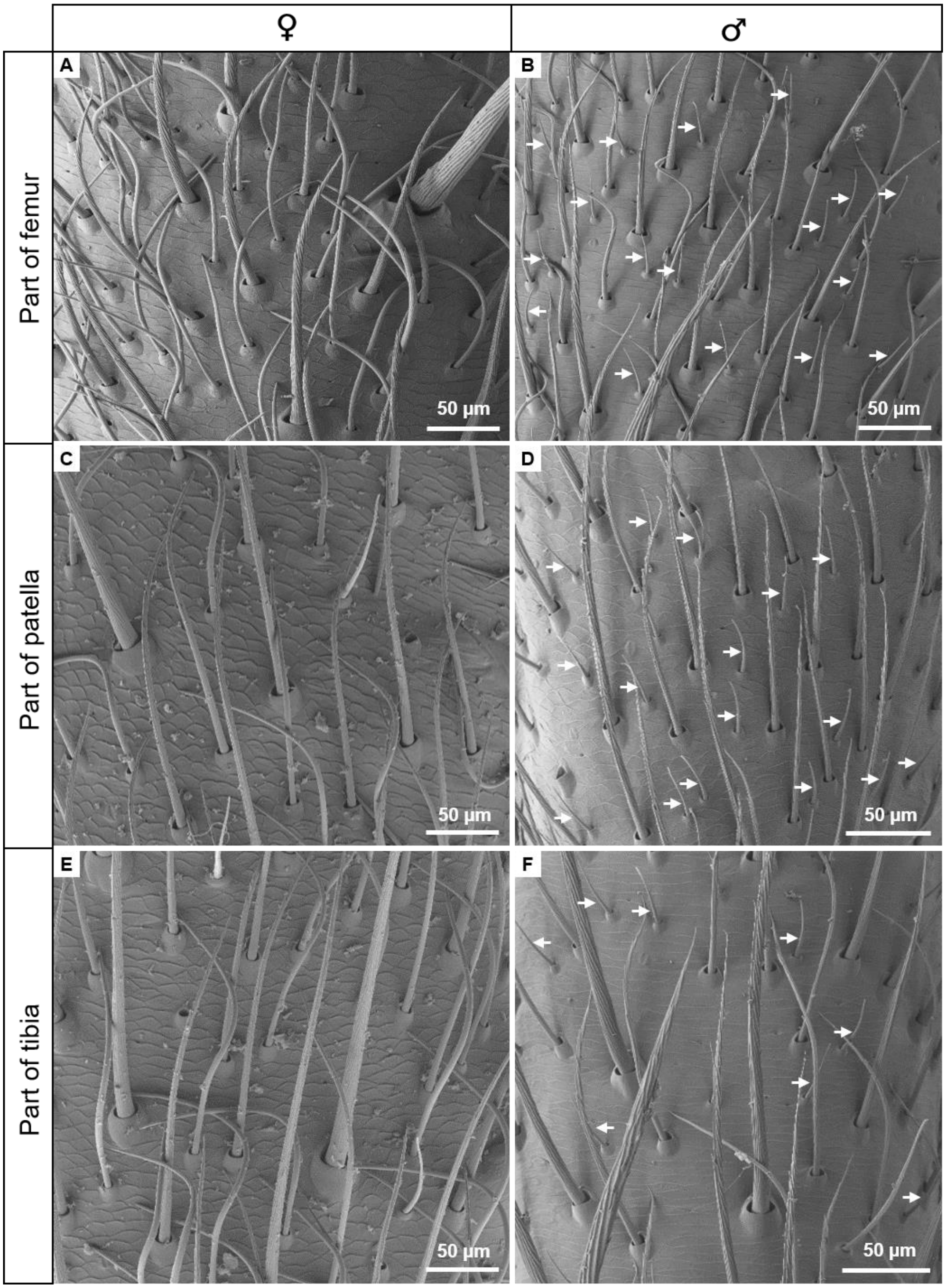

Fig. S3. SEM micrographs of representative areas of different leg podomeres (segments) of female and male *Argiope bruennichi*. Wall-pore sensilla are absent in females (**A**, **C**, **E**) but present in males (white arrows) (**B**, **D**, **F**).

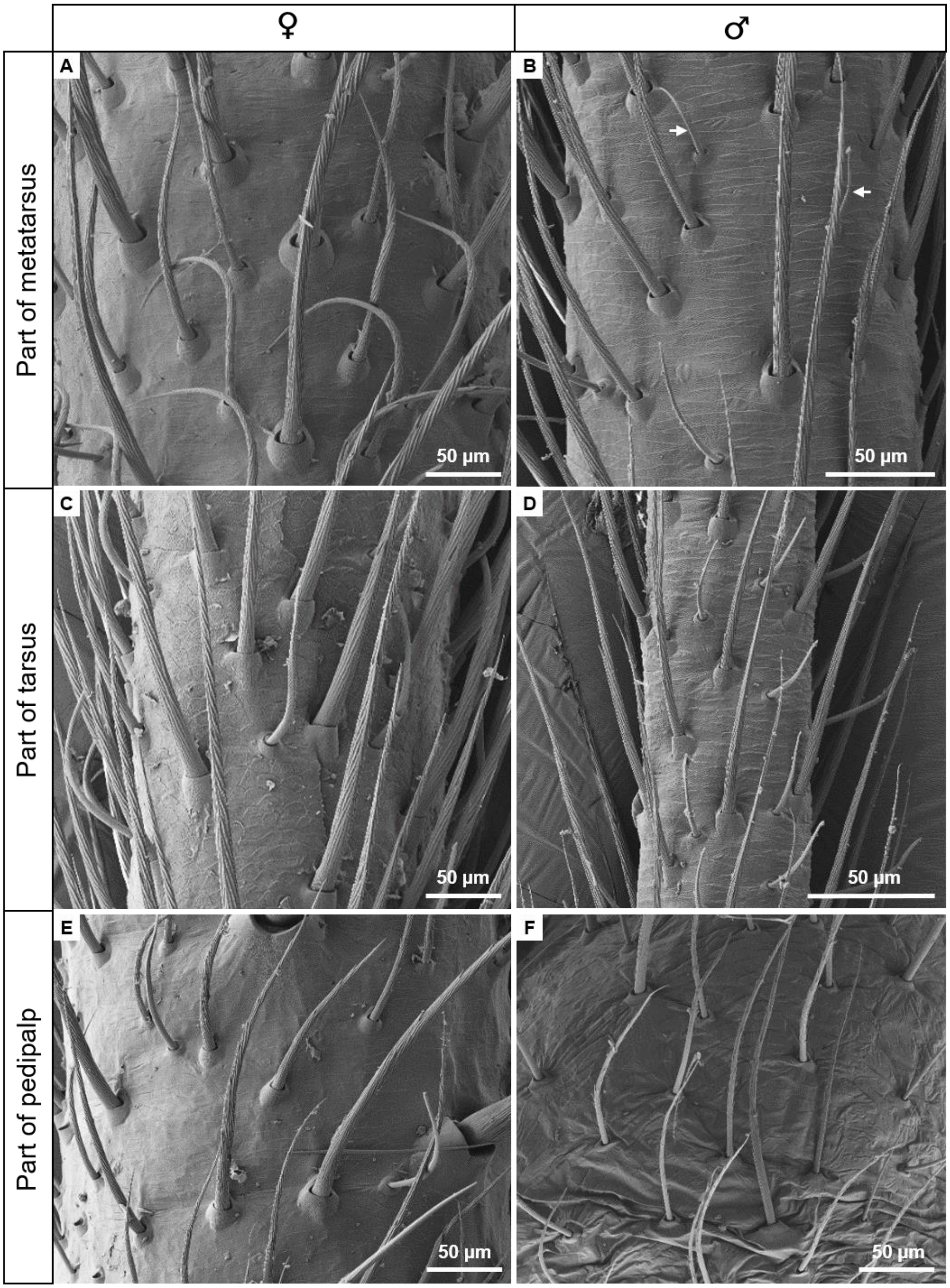

Fig. S4. SEM micrographs of representative areas of different leg podomeres of female and male *Argiope bruennichi*. Wall-pore sensilla are absent in females (**A**, **C**, **E**), tarsus and pedipalp of males (**D** and **F**), and present on metatarsus of males (white arrows) (**B**).

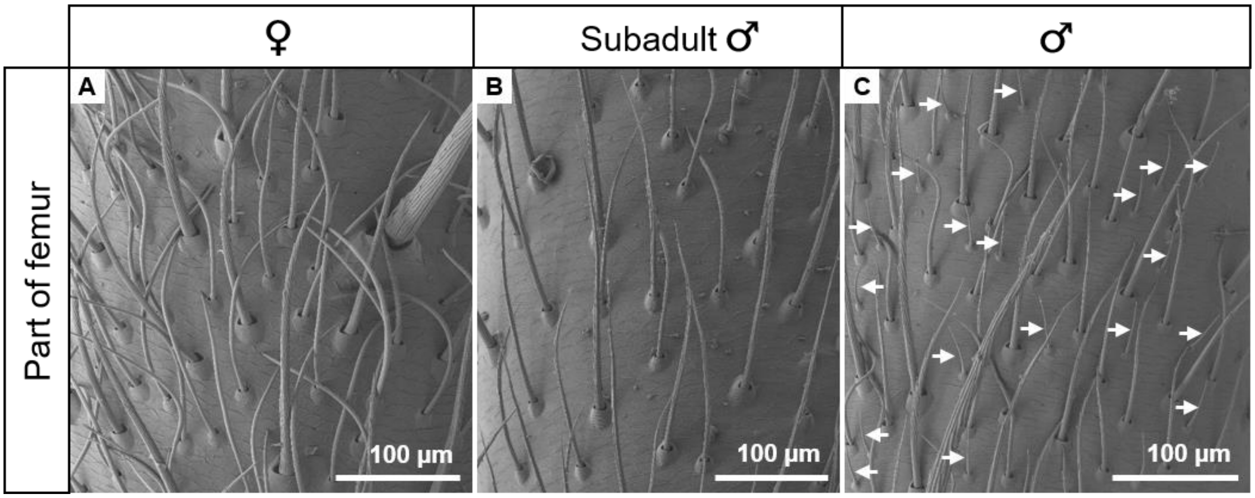

Fig. S5. SEM micrographs of representative areas of femur of adult female, subadult male, and adult male *Argiope bruennichi*. (**A**) An exemplary region of femur of adult female shows absence of wp-sensilla. (**B**) Corresponding region of femur of subadult male also shows absence of wp-sensilla. (**C**) Corresponding region of femur of adult male shows presence of wp-sensilla (white arrows).

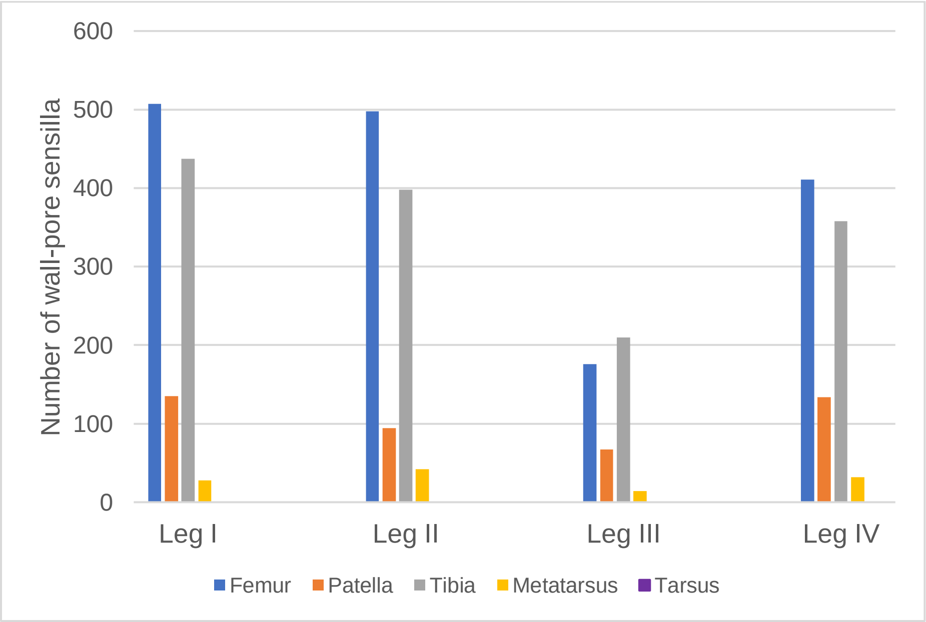

Fig. S6. Number of wall-pore sensilla on each podomere of the walking legs of an *Argiope bruennichi* male. Note that the tarsus does not have wall-pore sensilla.

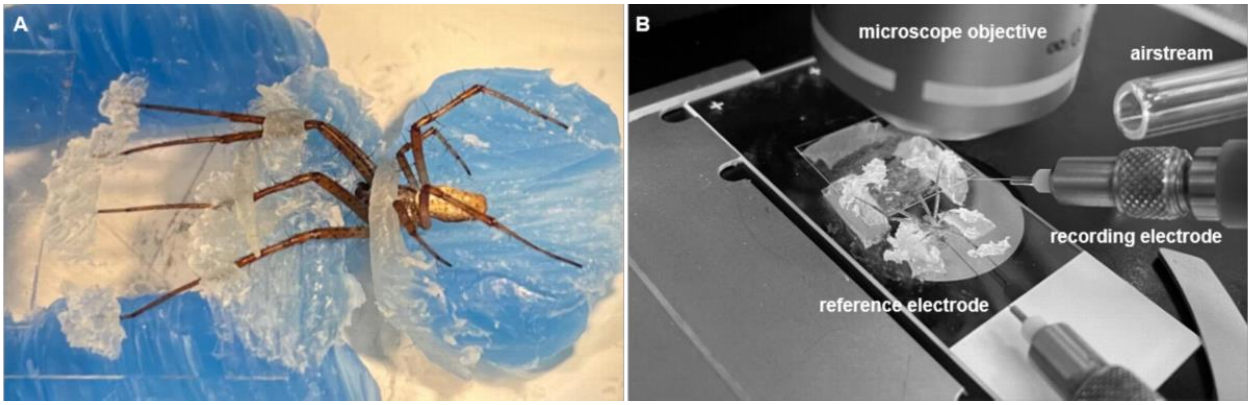

Fig. S7. Set-up of electrophysiological recording. (A) Mounting of an adult male *Argiope bruennichi*. A piece of cover glass was fixed on a glass slide with dental wax. The walking legs to be tested were gently fixed on the cover glass support, and the rest of walking legs and other body parts were fixed on the slide. **(B)** For single sensillum recording, a tungsten reference electrode was inserted into the posterior end of the spider´s opisthosoma. A tungsten recording electrode was inserted into the base of a wall-pore sensillum to contact the olfactory sensory neurons inside. A constant flow of charcoal-filtered and humidified air carrying the volatile stimulus was directed to the podomere that contained the target sensillum.

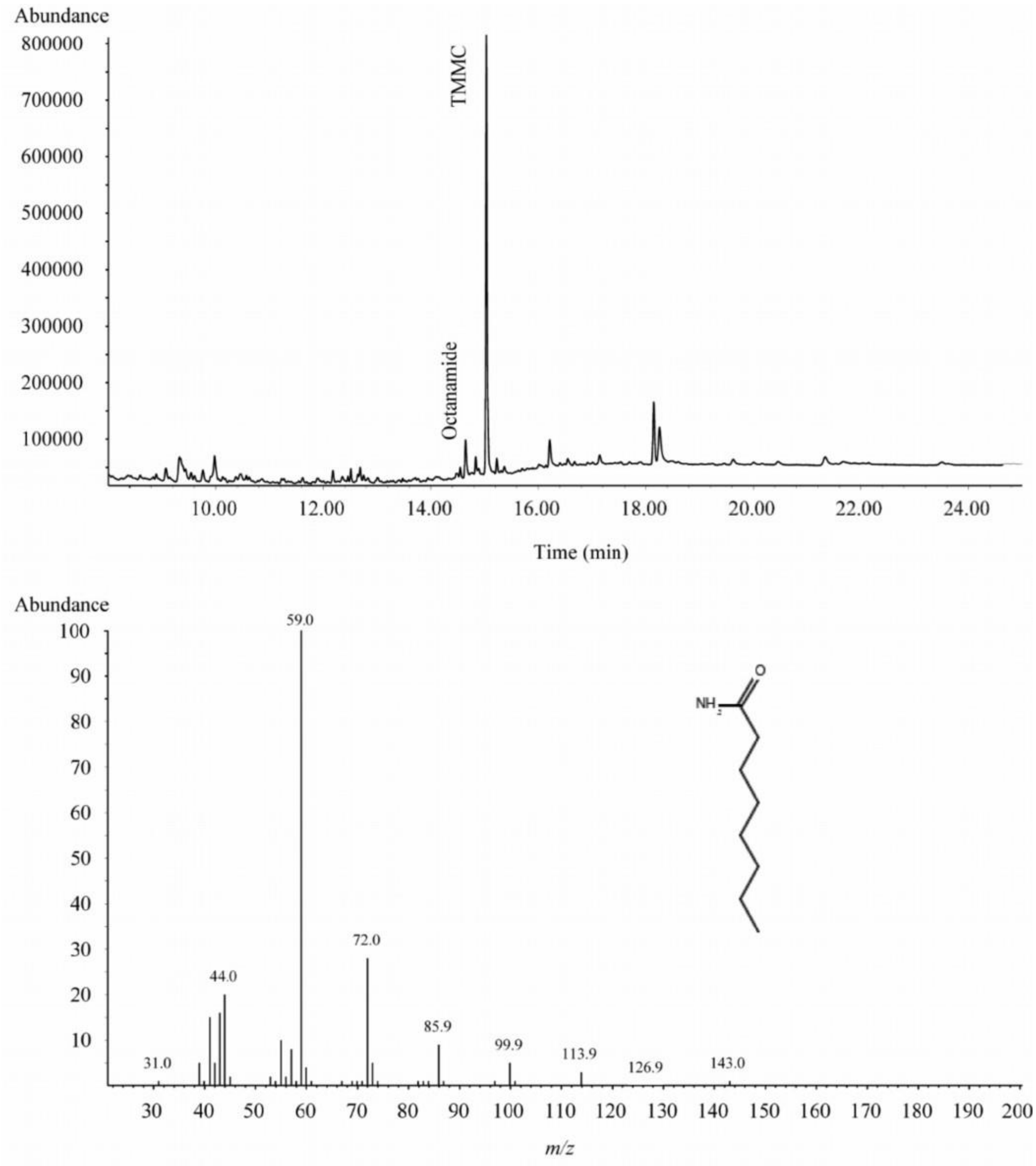

Fig. S8. Gas chromatography and mass spectrometry analysis of silk extract of an adult female *Argiope bruennichi*. The minor component eluted in the chromatograph (top panel) at the retention time 14.7 min showed identical retention time and mass spectrum (bottom panel) as the standard compound octanamide. The insert in the bottom panel shows the chemical structure of this compound. The dominant peak eluted at 15.1 min was identified as the sex pheromone compound (2*R*,3*S*)-trimethyl methylcitrate (TMMC). The total amount TMMC in the silk extract was quantified as approximately 4.9 µg.

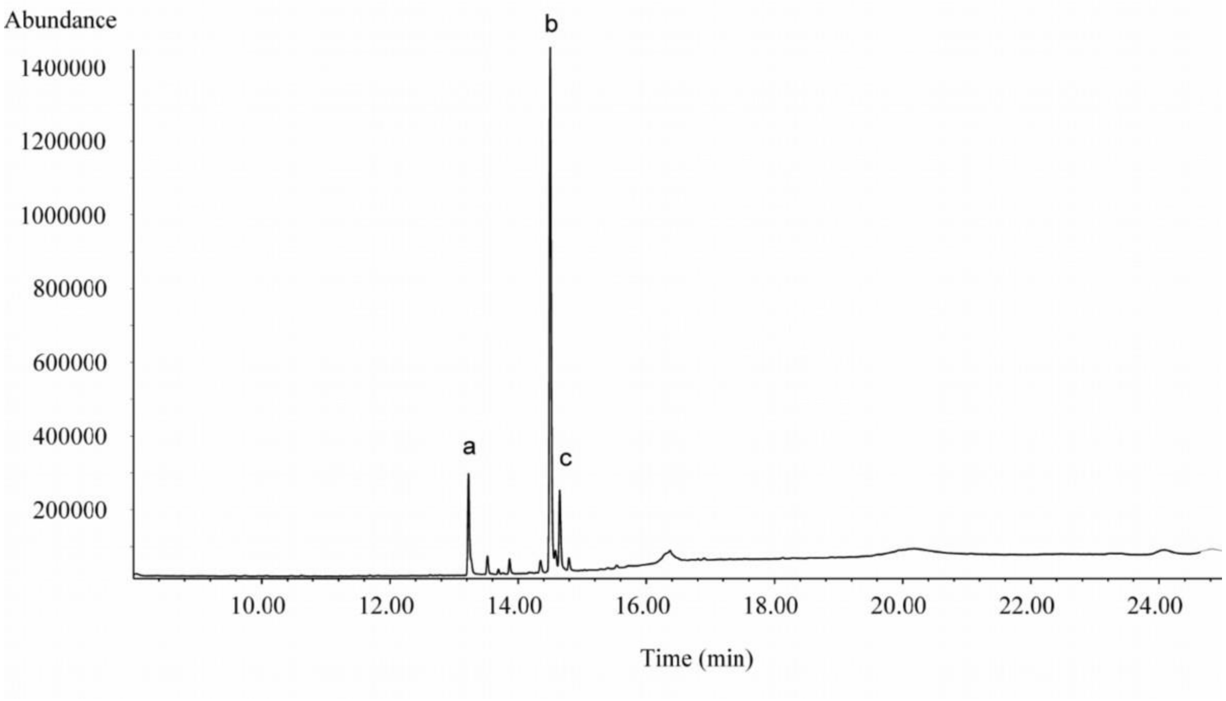

Fig. S9. Total ion chromatogram of the degraded products of tetradecyl (2*S*,4*S*)-2,4-dimethylheptadecanoate, a representative cuticle wax ester that contains the *Argiope* characteristic 2,4-dimethyl group. a, tetradecanol; b, methyl 2,4-dimethylheptadecanoate; c, isomer of methyl 2,4-dimethylheptadecanoate.

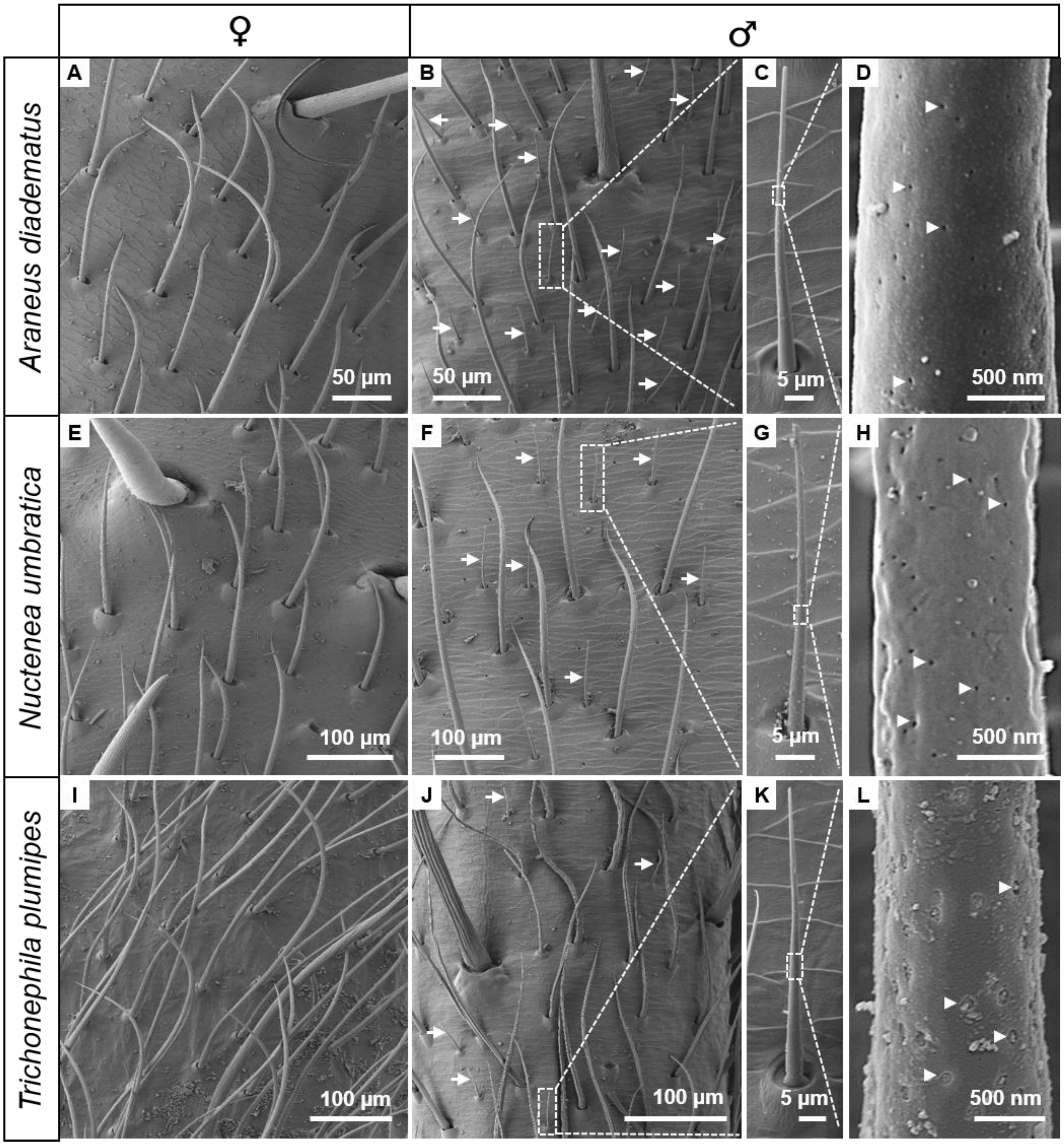

Fig. S10. Comparative approach I. FE-SEM micrographs of representative areas on leg podomeres of female and male spiders from different species and families. Wall-pore sensilla are marked by white arrows. (**A** to **D)** Femur of *Araneus diadematus* (Araneidae) (**E** to **H**) Femur of *Nuctenea umbratica* (Araneidae). (**I** to **L)** Tibia of *Trichonephila plumipes* (Araneidae, Nephilinae). (**D, H** and **L)** Arrowheads mark pores in the shaft wall of the sensillum.

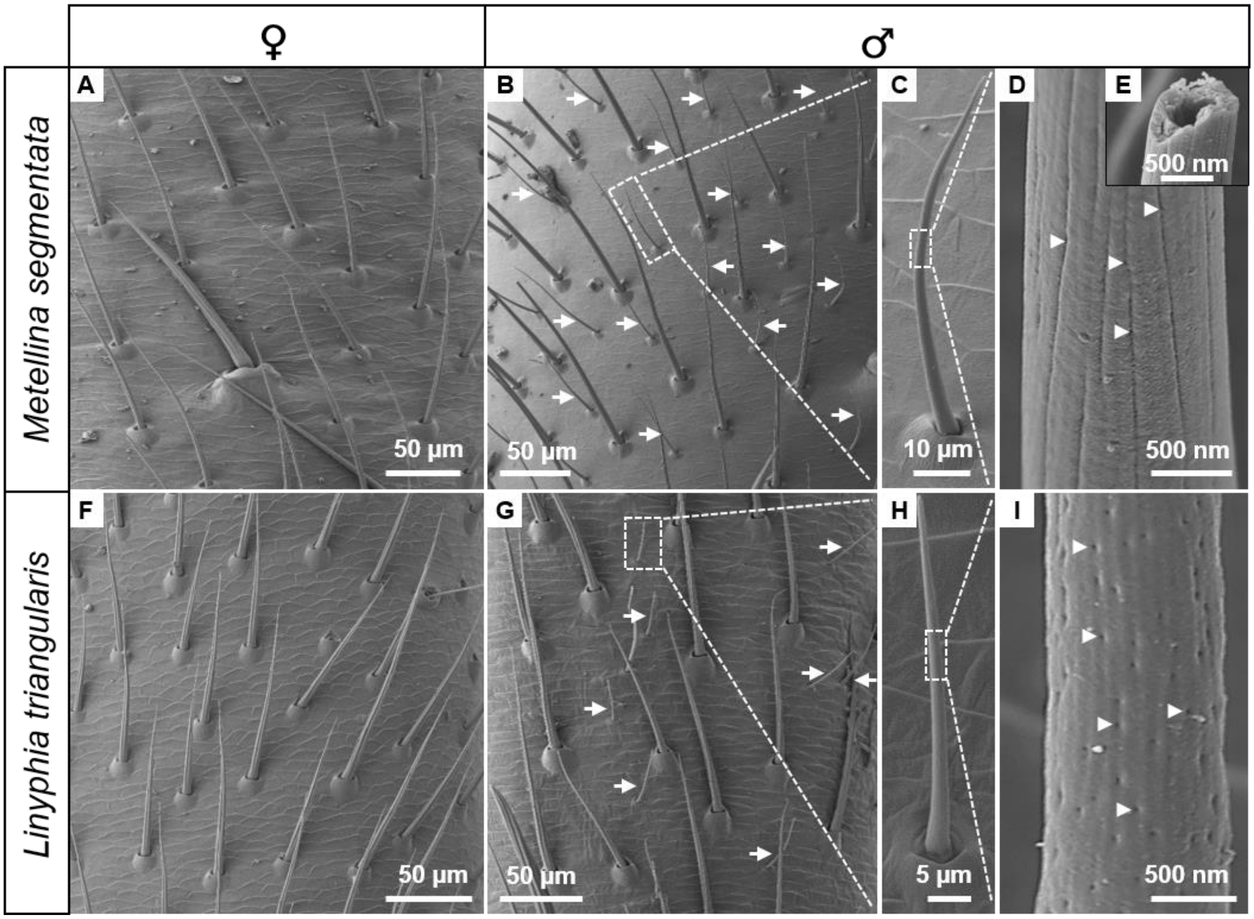

Fig. S11. Comparative approach II. FE-SEM micrographs of representative areas on leg podomeres of female and male spiders from different species and families. Wall-pore sensilla are marked by white arrows. (**A** to **E**) Femur of *Metellina segmentata* (Tetragnathidae). (**E)** A broken sensillum showing hollow centre, indicative of a chemosensillum. (**F** to **I**) Tibia of *Linyphia triangularis* (Linyphiidae). (**D** and **I**) Arrowheads mark pores in the shaft wall of the sensillum.

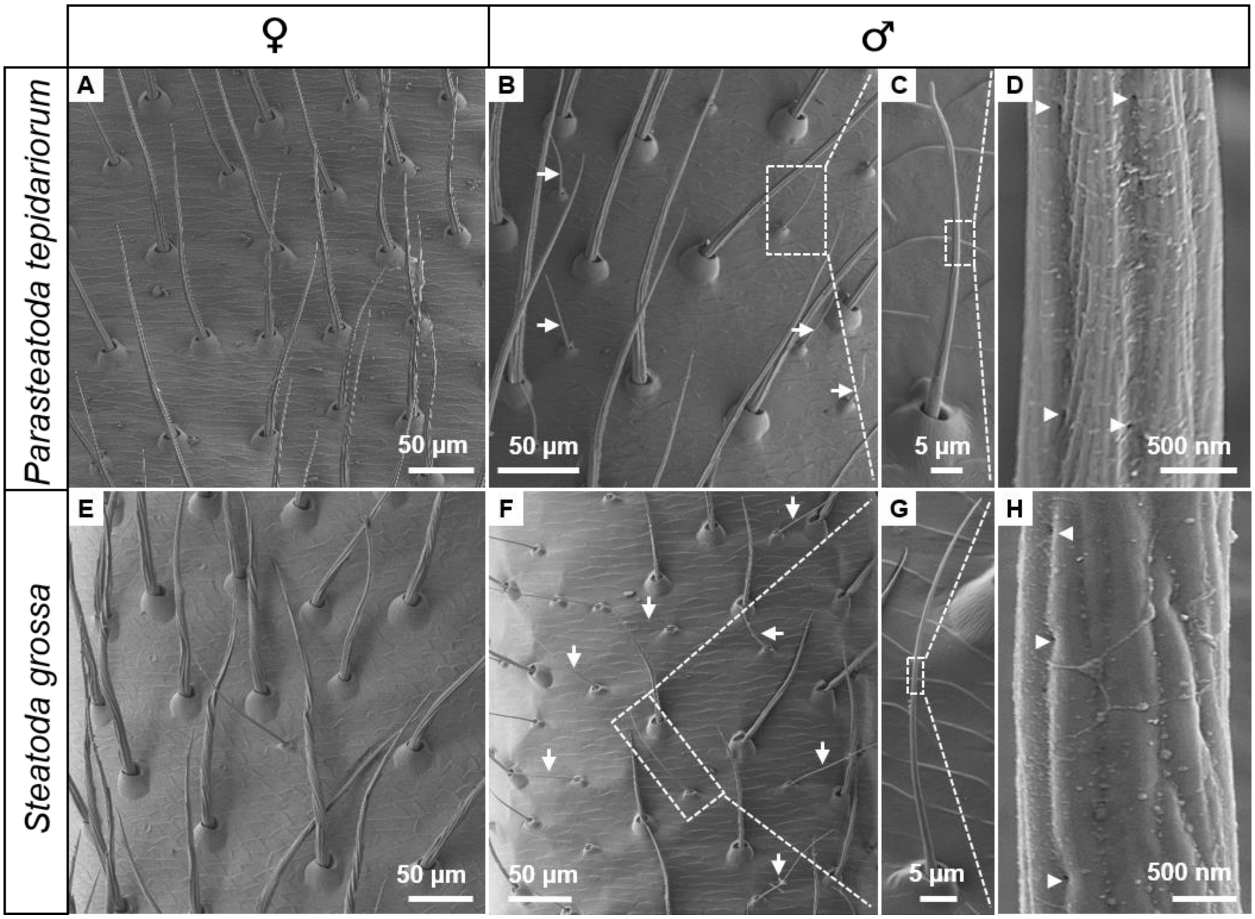

Fig. S12. Comparative approach III. FE-SEM micrographs of representative areas on leg podomeres of female and male spiders from 2 species of Theridiidae. Wall-pore sensilla are marked by white arrows. (**A** to **D**) Femur of *Parasteatoda tepidariorum* (Theridiidae). (**E** to **H**) Femur of *Steatoda grossa* (Theridiidae). (**D** and **H**) Arrowheads mark pores in the shaft wall of the sensillum.

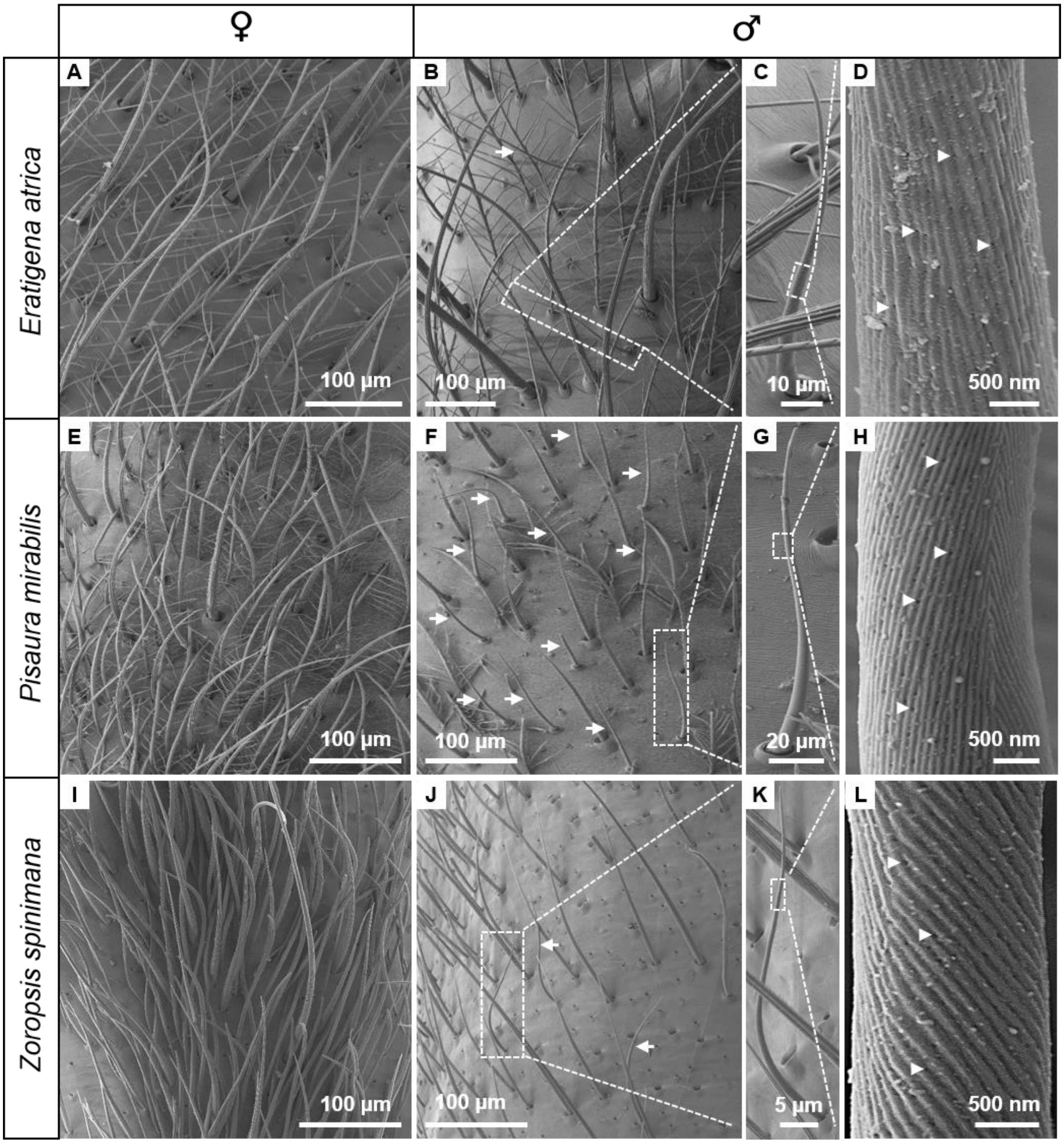

Fig. S13. Comparative approach IV. FE-SEM micrographs of representative areas on leg podomeres of female and male spiders from different species and families. Wall-pore sensilla are marked by white arrows. (**A** to **D**) Femur of *Eratigena atrica* (Agelenidae). (**E** to **H**) Femur of *Pisaura mirabilis* (Pisauridae). (**I** to **L**) Femur of *Zoropsis spinimana* (Zoropsidae). (**D, H** and **L**) Arrowheads mark pores in the shaft wall of the sensillum.

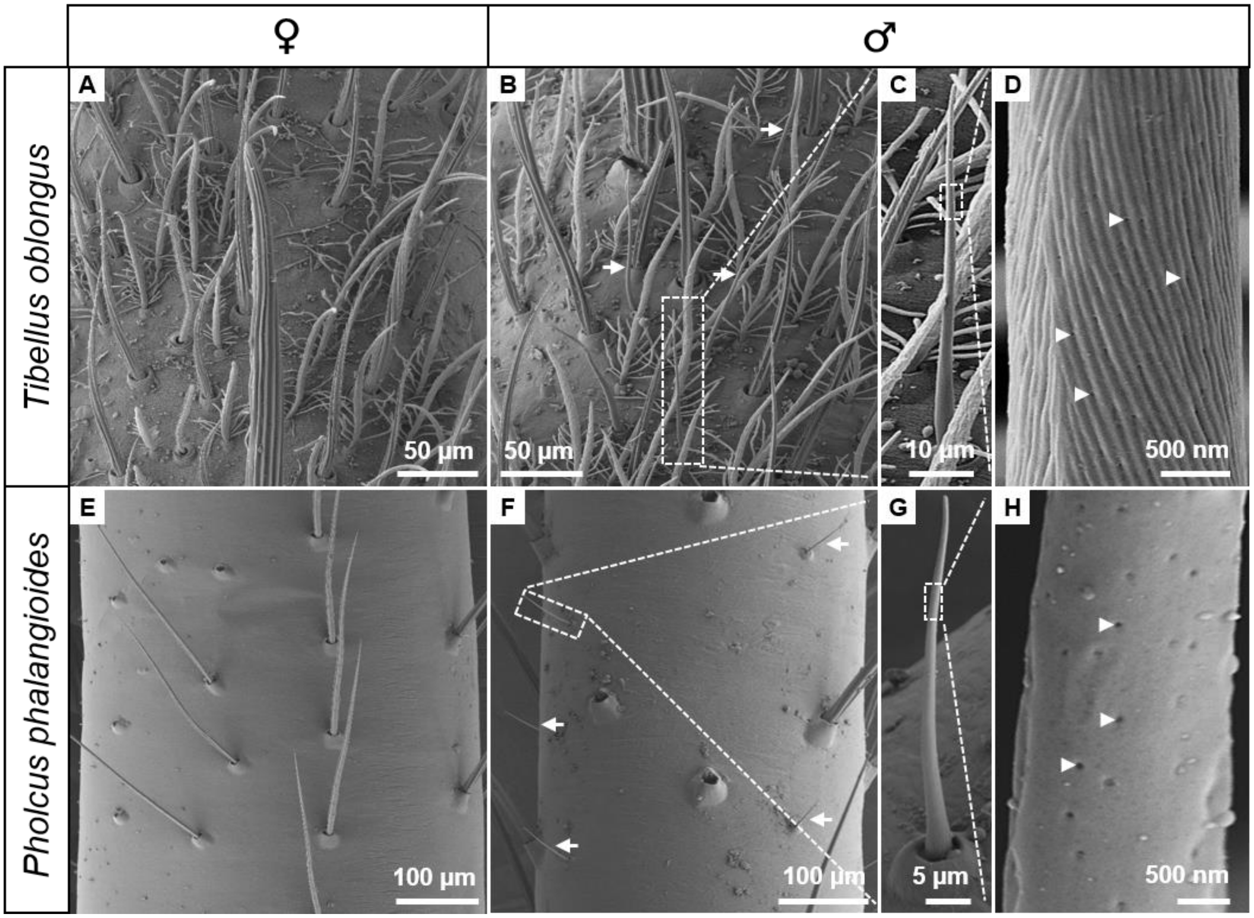

Fig. S14. Comparative approach V. FE-SEM micrographs of representative areas on leg podomeres of female and male spiders from different species and family. (**A** to **D**) Tibia of *Tibellus oblongus* (Philodromidae). (**E** to **H**) Tibia of *Pholcus phalangioides* (Pholcidae). The sensilla in this species very easily break off during handling. (**D** and **H**) Arrowheads mark pores in the shaft wall of the sensillum.

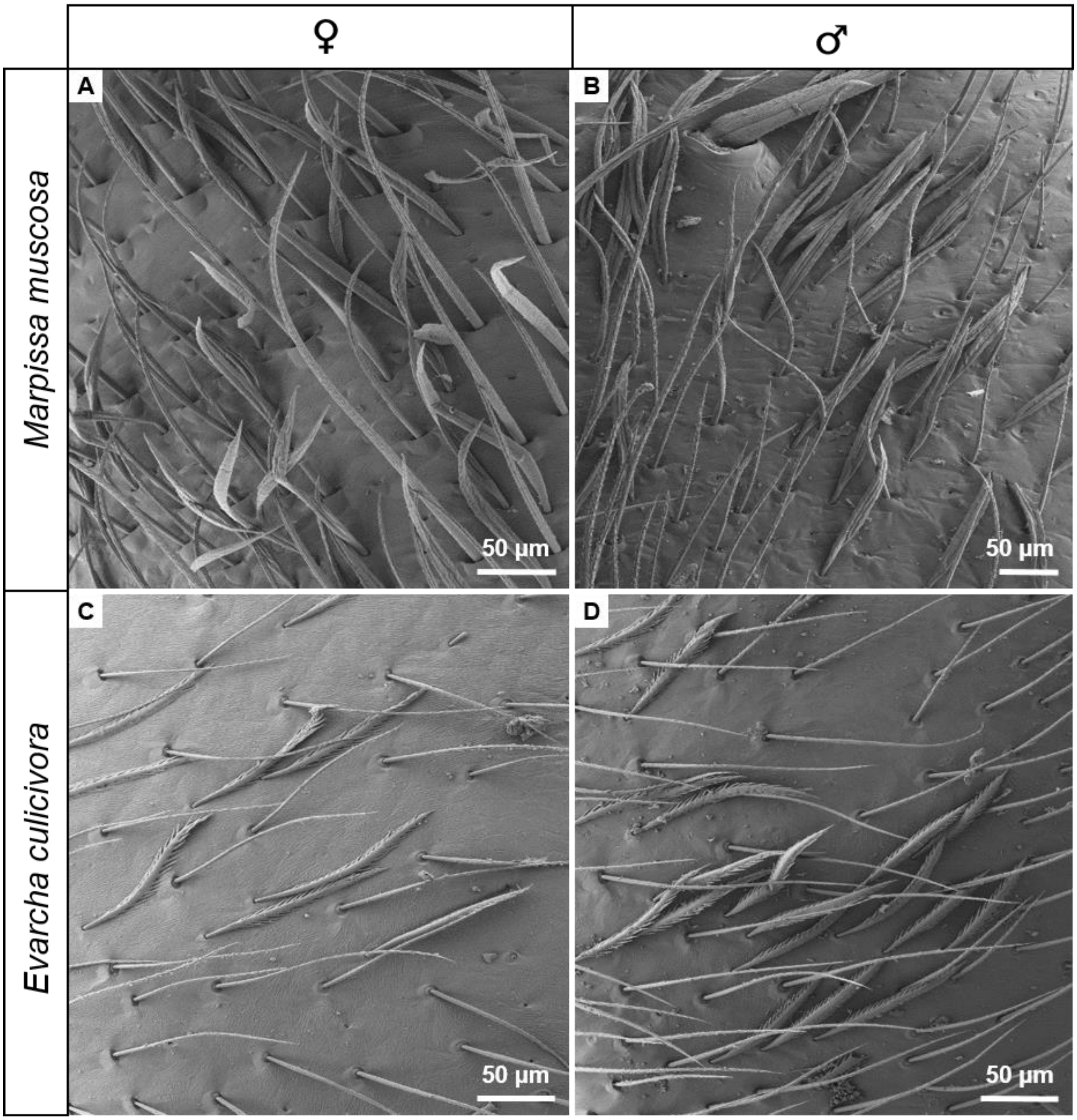

Fig. S15. Comparative approach VI. FE-SEM micrographs of representative areas on leg podomeres of female and male spiders from two species of salticids. No wall-pore sensilla were found. (**A** and **B**) Femur of *Marpissa muscosa* (Salticidae). (**C** and **D**) Femur of *Evarcha culicivora* (Salticidae).

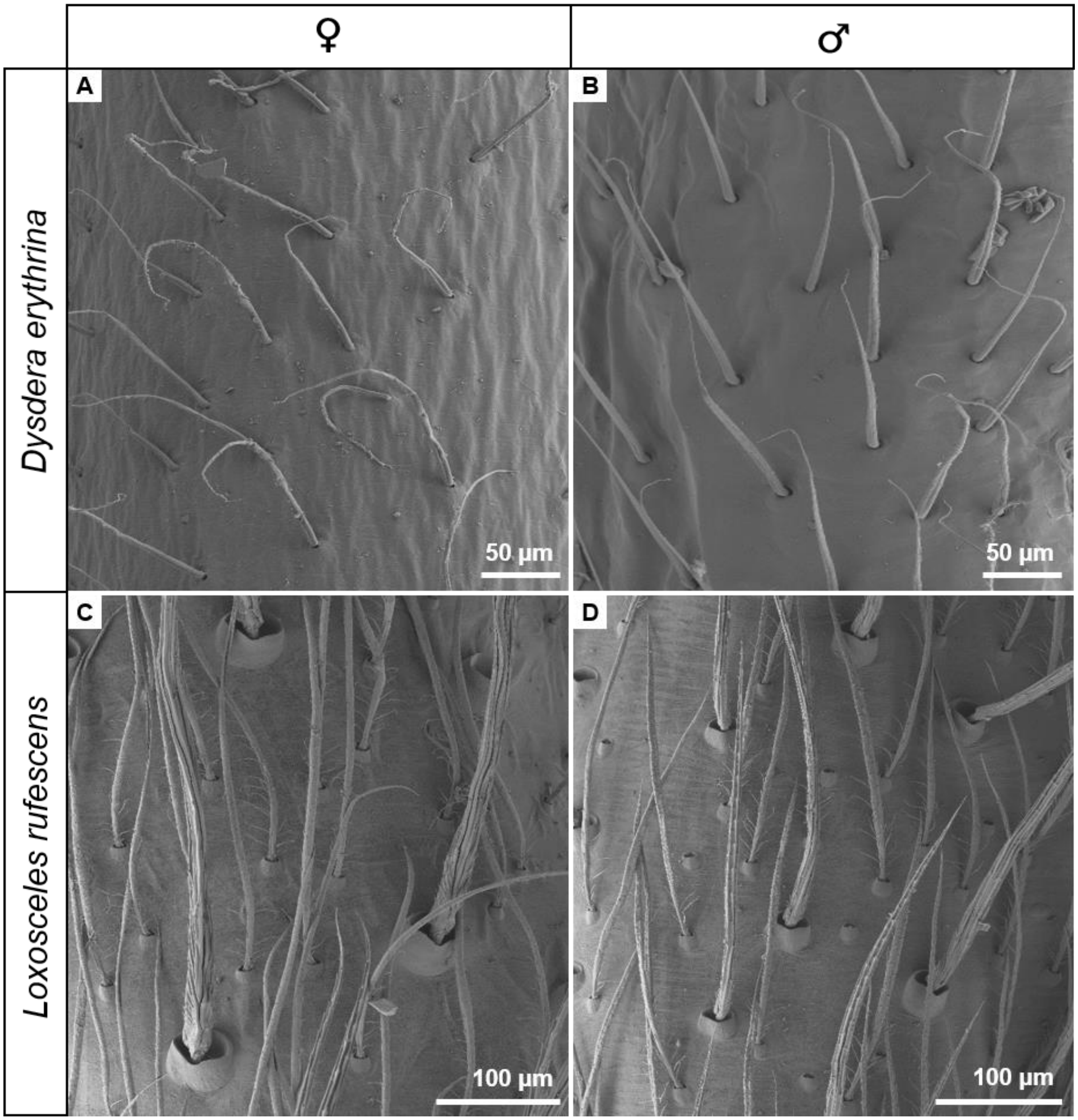

Fig. S16. Comparative approach VII. FE-SEM micrographs of representative areas on leg podomeres of female and male spiders from different species and families. No wall-pore sensilla were found. (**A** and **B**) Femur of *Dysdera erythrina* (Dysderidae). (**C** and **D**) Femur of *Loxosceles rufescens* (Sicariidae).

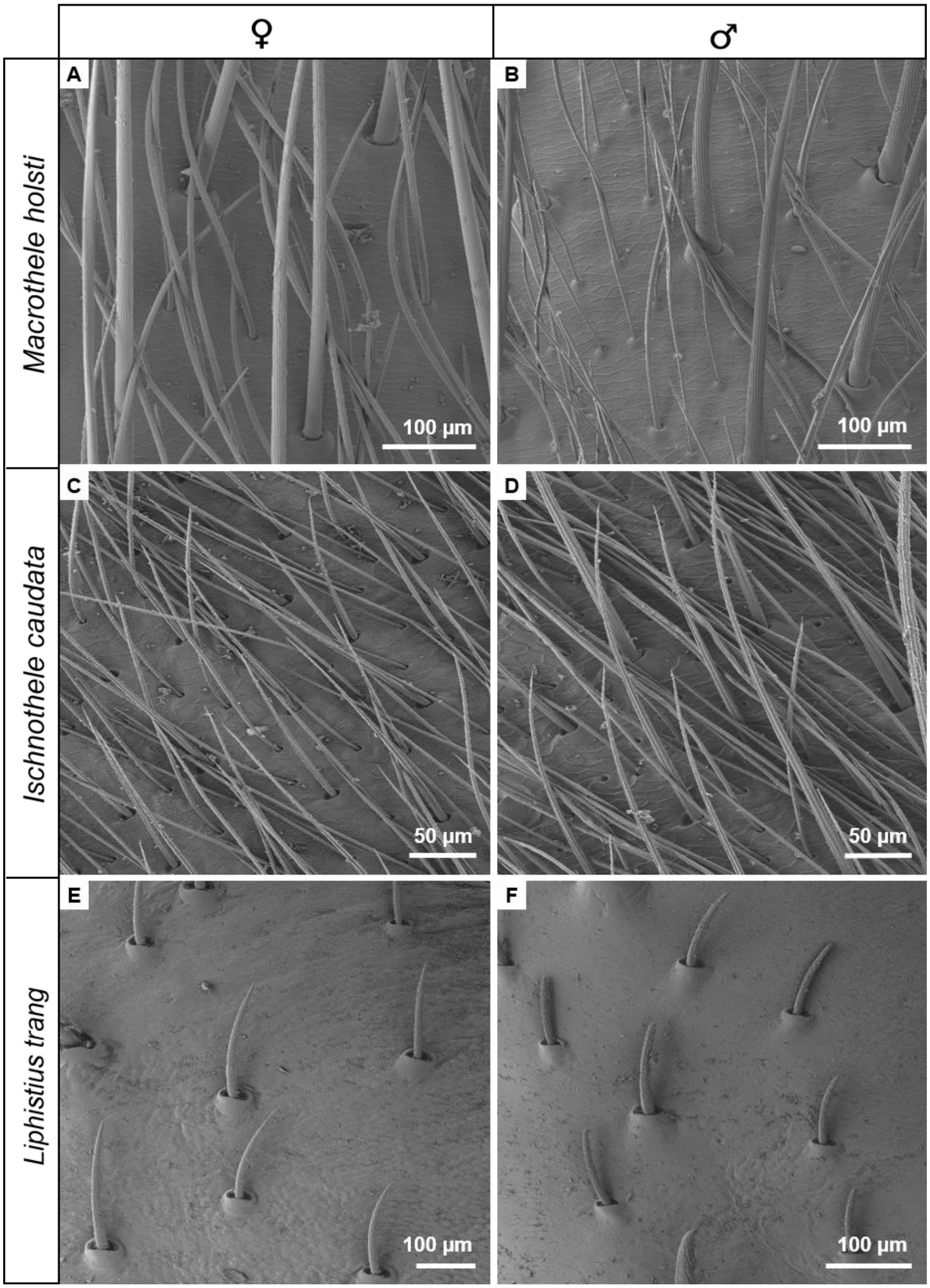

Fig. S17. Comparative approach VIII. FE-SEM micrographs of representative areas on leg podomeres of female and male spiders from different species and families. No wall-pore sensilla were found. (**A** and **B**) Femur of *Macrothele holsti* (Macrothelidae). (**C** and **D**) Femur of *Ischnothele caudata* (Ischnothelidae). (**E** and **F**) Femur of *Liphistius trang* (Liphistiidae).

Table S1. Probability in % that body appendages of *Argiope bruennichi* male and female come into contact with different substrates during walking (6 males and 6 females walked on 5 substrates each; flat surface, artificial leaves, stick, grass, female´s web. N= 30 videos for males and N= 30 total videos for females), mating (N=6 mating trials, using 6 mating pairs) and prey capturing by the female (N=6 trials, using 6 females. Note that males do not capture prey); specified for the segments of the walking legs (femur to tarsus) and the pedipalps (n.a.= lacks metatarsus). I-IV: 1st to 4th walking leg. fe: femur, pt: patella, ti: tibia, mt: metatarsus, ta: tarsus.

		**Male**				**Female**			
		**fe**	**pt-ti**	**mt**	**ta**	**fe**	**pt-ti**	**mt**	**ta**
**Walking**	Leg I	0	0	10	100	0	0	16	100
	Leg II	0	0	10	100	0	0	10	100
	Leg III	0	0	13	100	0	0	3	100
	Leg IV	0	0	10	100	0	0	16	100
	pedipalp	0	0	n.a.	20	0	0	n.a.	70
**Mating**	Leg I	0	0	66	100	0	0	33	100
	Leg II	0	0	50	100	0	0	33	100
	Leg III	0	0	16	66	0	0	16	100
	Leg IV	0	0	16	83	0	0	16	100
	pedipalp	0	0	n.a.	100	0	0	n.a.	16
**Prey capturing**	Leg I	n.a				0	0	0	100
	Leg II					0	0	0	100
	Leg III					0	0	0	100
	Leg IV					0	0	0	100
	pedipalp					0	0	n.a	100

Movie S1 (separate file): Contact and non-contact areas on body appendages of a male *A. bruennichi*. Slow-motion footage (500 frame per second) of a male walking on a horizontal skewer. The video highlights that only the distal leg segments make contact with the surface.

Movie S2 (separate file): Electrophysiological responses to the female sex pheromone recorded from a wall-pore sensillum on the leg of a male spider *Argiope bruennichi*. The pheromone compound (2*R*,3*S*)-trimethyl methylcitrate was applied as stimulus on the filter paper in a test tube and puffed into the air flow with different doses, i.e., 20 ng, 200 ng and 2000 ng sequentially at 7.5 s, 22 s and 41 s for 500 ms. The amplitudes of the potentials are given in mV. Three neuron cells (referring to pattern a+b+d in Fig. 4E) were observed based on their spontaneous potential. Under the high dose, the pheromone responding neuron was adapted for a few seconds followed by a quick recovery. A 10x gain DC probe and an IDAX4 controller (Syntech) were used to capture and amplify the signals using Autospike software (Synthetic, V.3.9, Buchenbach, Germany).
